# Performance of Hybrid and All-Inorganic Perovskite Direct X-Ray Imagers: Surpassing Commercial Standards

**DOI:** 10.1002/admt.202501491

**Published:** 2025-12-21

**Authors:** Brandon Dunham, Shariar Motakef, Amlan Datta

**Affiliations:** CapeSym, Inc., 6 Huron Drive, Natick, MA 01760, USA

**Keywords:** detector, flat panel, perovskite, resolution, X-ray Imaging

## Abstract

Solution-processed metal halide perovskites have rapidly emerged as promising candidates for direct X-ray imaging, yet fundamental comparisons between hybrid and all-inorganic systems in flat panel architectures remain missing. A systematic study of perovskite X-ray imagers (PeroXIs) based on methylammonium lead iodide and cesium lead bromide films integrated onto identical active pixel arrays is presented. Fully inorganic imagers demonstrate excellent performance characteristics, achieving high spatial resolution, contrast, sensitivity, and operational stability. While hybrid perovskites also show promising results, the all-inorganic systems exhibit characteristics that may be particularly advantageous for repeatable performance and therefore commercial deployment. Crucially, the all-inorganic PeroXI overcomes the long-standing trade-off between spatial resolution and detection efficiency that has limited X-ray detection technologies for decades, achieving high performance on both fronts. The results establish solution-processed inorganic perovskites as a scalable, high-performance platform capable of surpassing commercial standards, opening a new pathway toward low-cost, high-resolution, and low-dose X-ray imaging across medical, industrial, and scientific domains.

## 1 | Introduction

Flat panel X-ray imagers (FPXIs) have revolutionized digital radiography over the past decades, finding widespread use across medical, industrial, and scientific applications. These devices enable high-resolution, real-time X-ray imaging for modalities such as digital radiography, fluoroscopy, tomosynthesis, image-guided radiation therapy, and cone beam computed tomography [1, 2]. Beyond medicine, FPXIs have become indispensable tools for non-destructive testing, cultural heritage investigations, materials science research, and security screening [3–5].

FPXIs can be broadly categorized into two types based on their X-ray detection mechanism: indirect and direct conversion. Indirect FPXIs dominate the current market, relying on scintillator materials like thallium-doped cesium iodide (CsI:Tl) or gadolinium oxysulfide (Gadox) to convert X-rays into visible light, which is then detected by photodiode arrays [6]. These systems benefit from high detective quantum efficiency (DQE), even at higher X-ray energies, due to the high atomic number of elements in scintillators. However, they suffer from inherent limitations in spatial resolution caused by optical crosstalk and light spread within the scintillator layer [7]. Additionally, scintillator afterglow can introduce artifacts in dynamic imaging scenarios [8].

In contrast, direct conversion FPXIs utilize semiconductor materials that directly convert X-ray photons into electrical charges [9]. This one-step process eliminates the intermediate light conversion step, potentially offering superior spatial resolution and faster response times compared to indirect systems [10]. However, the widespread adoption of direct conversion FPXIs has been hindered by limitations in current semiconductor materials. Amorphous selenium (a-Se) is the most commercially successful direct conversion material to date, offering ease of large-area deposition and good spatial resolution [11]. However, its low atomic number results in poor X-ray absorption at energies above 30 keV, limiting its use primarily to mammography [12]. Additionally, a-Se requires high electric fields (10-40 V/μm) for efficient charge collection, which can lead to stability issues over time [13]. Crystalline semiconductors like cadmium telluride (CdTe) and cadmium zinc telluride (CZT) offer excellent X-ray absorption and charge transport properties, enabling high-resolution imaging even at higher X-ray energies [14]. However, the challenges and costs associated with growing large, uniform CdTe or CZT crystals and integrating them with readout electronics have restricted their use to niche applications like computed tomography [15].

In recent years, metal halide perovskites (MHPs) have gained significant attention as promising candidates for next-generation direct conversion X-ray detectors [16, 17]. These materials, characterized by the general formula ABX_3_, where A is typically an organic or inorganic monovalent cation, B is a divalent metal cation, and X is a halide anion, possess a unique combination of properties that make them highly suitable for X-ray detection. One of the key advantages of MHPs, particularly those incorporating high atomic number elements such as lead (Pb) and cesium (Cs), is their capacity for efficient X-ray absorption [11, 18]. This high attenuation capability enables the detection of X-rays across a broad energy range comparable, and occasionally superior to those of common commercial detectors like CdTe and CsI (Figure S1), enhancing the overall detector performance. Additionally, MHPs exhibit large mobility-lifetime (μ*τ*) products [19], which facilitate efficient charge collection by allowing photogenerated carriers to travel longer distances without recombining or being trapped. This characteristic is crucial for maintaining high sensitivity and spatial resolution in imaging applications. Another notable feature of MHPs is their low ionization energy [20], which contributes to their high sensitivity in X-ray detection by enabling the material to generate charge carriers with minimal energy input. Furthermore, these materials are solution-processable, which opens up the possibility for cost-effective and scalable large-area fabrication methods [17, 21]. This property is particularly valuable in the production of large-area FPXIs, where traditional materials can be cost-prohibitive or difficult to manufacture on a large scale. MHPs also offer compositional flexibility, allowing for the tuning of their bandgap and other material properties. This flexibility enables the optimization of MHPs for specific X-ray detection applications by adjusting their absorption spectrum and electronic characteristics to match the requirements of different imaging modalities [22]. Finally, MHPs have been demonstrated to be one of the only materials capable of detecting X-rays at submicron film thicknesses [23–26]. This has widely been attributed to a phenomenon called trap-mediated photoconductive gain, enabling very good sensitivity despite limited X-ray absorption from the thin MHP film. By exploiting this remarkable material property, it would be possible to manufacture thin-film MHP X-ray imagers on flexible substrates, which is com for CZT and a-Se based detectors. Collectively, these attributes position MHPs as highly versatile and efficient materials for the future of X-ray imaging technologies.

Two primary MHP compositions, methylammonium lead iodide (CH_3_NH_3_PbI_3_ or MAPI) and cesium lead bromide (CsPbBr_3_ or CPB), have garnered particular attention for X-ray detection due to their distinct properties and potential for integration into high-performance detectors. MAPI, a hybrid organic-inorganic material, is known for its optimal bandgap of approximately 1.5 eV (used in highly efficient MAPI-based perovskite solar cells) and a large μ*τ* product. These characteristics contribute to its impressive X-ray sensitivity, which can exceed 10^4^ μC Gy_air_^−1^ cm^−2^ in both single crystal and polycrystalline forms [27, 28]. However, MAPI faces challenges related to long-term stability and robustness. The organic methylammonium component is prone to ion migration and moisture sensitivity, leading to degradation in performance over time. This instability has driven research toward finding more robust alternatives that maintain MAPI’s high sensitivity but offer better durability [29, 30], such as formamidinium (FA) [24], guanidinium (GA) [31], or mixed cation (MAFA, CsFA, or CsFAGA) [32] based hybrid perovskites (where the larger organic cations offer enhanced structural stability to the crystal lattice) and fully inorganic perovskites. One such fully inorganic perovskite, CPB, has a wider bandgap of about 2.3 eV. This composition offers enhanced environmental stability, as its fully inorganic nature inherently reduces sensitivity to moisture and mitigates ion migration issues, thus making CPB-based detectors more stable over prolonged periods of operation [33]. Recent studies have shown that CPB detectors can achieve X-ray sensitivities exceeding those of MAPI [34, 35]. Furthermore, the stability and reproducibility of CPB under various operational conditions position it as a strong candidate for practical, long-term applications. Despite these advantages, challenges remain in scaling both MAPI and CPB into full-scale FPXIs. These include achieving uniform large-area deposition, ensuring good contact with the underlying readout electronics, and maintaining performance over extended periods under operational conditions [36].

To date, various manufacturing methods have been used to fabricate larger area perovskite-based sensors for X-ray detection, such as fast tableting, spray coating, and blade coating. The fast tableting technique is capable of quickly and repeatably producing precise and uniform thick films or wafers. Li et al. employed fast tableting to make a 1.33 cm^2^ 4-fluorophenethylammonium bismuth iodide 2D perovskite wafer with good charge collection and suppressed ion migration that yielded a sensitive response to hard X-rays operated at a tube voltage of 120 kVp with the lowest detectable dose rate of 30 nGy_air_ s^−1^ [37]. Nevertheless, it can be difficult to integrate the films or wafers produced via this method with large-area thin film transistor (TFT) backplanes while maintaining good sensor-to-substrate adhesion. Spray coating, on the other hand, offers material-efficient, high-throughput deposition of thick films with good coverage on substrates like TFT backplanes. Precise control over film thickness and quality can be difficult, however, since environmental factors (e.g., temperature and humidity), solution properties (e.g., viscosity and purity), and spraying parameters (e.g., nozzle selection and distance from substrate) greatly influence the spray process. After carefully optimizing a pneumatic spray-coating technique and polishing the resulting film surface after deposition, He et al. fabricated 980-μm-thick Cs_0.05_FA_0.9_MA_0.05_PbI_3_ perovskite sensors for CT imaging that showed a low noise-equivalent dose of 153 pGy_air_ at 59.3 keV [38]. Here, we chose to employ a simple blade-coating strategy due to its scalability, ability to coat large areas, and facile integration with TFT backplanes for solution-processible perovskites. Optimization of our perovskite solution, coating speed, and blade gap enabled us to achieve high-quality and conformal 200-μm-thick perovskite films with remarkable X-ray imaging properties.

Several pioneering works have demonstrated the feasibility of MHP-based FPXIs. In 2017, Kim et al. reported the first large-area (10 × 10 cm^2^) MAPI-based FPXI fabricated by blade-coating, achieving a spatial resolution of 3.1 lp/mm [21]. Deumel et al. later improved upon this, reaching 3.4 lp/mm resolution using a soft-sintered MAPI approach [39]. For CPB, Li et al. recently demonstrated a perovskite-heterostructure X-ray imager with a high resolution of 4.6 lp/mm [35].

Despite these pioneering demonstrations, a critical gap remains in our understanding of MHP-based X-ray imaging: no study has yet provided a systematic, side-by-side comparison of hybrid versus all-inorganic perovskite compositions under identical device architectures and testing conditions. This knowledge gap has hindered the ability to make evidence-based decisions about optimal material selection for commercial development. Here, we address this fundamental challenge by presenting the first comprehensive, controlled comparison between MAPI-based and CPB-based FPXIs. Our systematic approach utilizes identical fabrication conditions for both systems - solution-processed polycrystalline MHP layers precisely controlled to certain thicknesses, deposited via blade-coating onto matching amorphous silicon (a-Si) active pixel array (APA) backplanes (512 × 512 pixels, 100 μm pitch). This carefully controlled experimental design not only eliminates variables that have historically complicated cross-study comparisons but also reveals the transformative potential of perovskite-based imaging. Most significantly, our CPB-based devices achieve unprecedented performance metrics that overcome the existing commercial standards, demonstrating superior spatial resolution (> 20 lp/mm), contrast, and detection efficiency. This breakthrough resolves a decades-old challenge in X-ray imaging: the fundamental trade-off between spatial resolution and detection efficiency that has constrained detector development in this field. By achieving exceptional resolution while maintaining high detection efficiency across a broad range of X-ray energies, our work establishes perovskite-based detectors as a revolutionary platform that surpasses the limitations of current commercial technologies. The insights gained from this controlled comparison, combined with the demonstration of performance that surpasses commercial standards, provide crucial guidance for the future development and commercialization of next-generation X-ray imaging technologies.

## 2 | Results and Discussion

### 2.1 | Single Pixel Detector Comparison: MAPI Versus CPB

To evaluate the potential of hybrid organic-inorganic and all-inorganic halide perovskites for X-ray detection applications, we conducted a comprehensive comparison between single-pixel detectors based on MAPI and CPB. The single pixel detectors operated as photodiodes with a bottom ITO contact and a top metal contact (e.g., Au, Pb, and Bi). Polymer-based charge-blocking layers, individually optimized for MAPI-based and CPB-based detectors, were inserted between the bottom ITO contact and the perovskite-based sensor layer to allow the device to operate in either hole-collecting or electron-collecting mode (depending on the polymer charge-blocking layer used). For this study, both the MAPI and CPB thick film sensor layers were fabricated through a blade coating process (see Methods for details) that yielded dense and compact films without any pinholes. The MAPI sensors were fabricated using commercial starting materials, ensuring consistency and reproducibility in our MAPI-based PeroXIs (M-PeroXIs). In contrast, the CPB sensors were produced using ultrahigh purity, spectroscopic-grade CPB single crystals as the precursor material (Figures S2 and S3 as well as [40]). These single crystals were grown in-house using an optimized Bridgman technique, which was crucial for achieving the required phase purity and material quality. X-ray diffraction (XRD) analysis of the CPB precursor and blade-coated sensor revealed characteristic peaks at approximately 15^◦^, 21^◦^, 30^◦^, 34^◦^, 37^◦^, and 43^◦^ 2*θ*, consistent with the stable low-temperature orthorhombic phase of CsPbBr_3_ with a Pnma space group (Figures S4 and S6). This high degree of structural consistency was crucial for maintaining uniform and reproducible electronic properties across different CPB-based PeroXIs (C-PeroXIs). Importantly, we observed that CPB materials not grown using our optimized technique often contained traces of the CsPb_2_Br_5_ tetragonal phase (Figure S5). The presence of this secondary phase introduces inconsistencies in detector performance due to its different electronic and physical properties. Additionally, we observed that precursor materials with multiple phases or higher levels of metallic impurities yielded nonhomogeneous precursor solutions that resulted in sensor films with large amounts of aggregates (Figure S7) that led to detectors with poor imaging capabilities (Figure S8). Our optimized Bridgman growth process effectively eliminated this impurity phase, resulting in highly pure orthorhombic CPB.

Our analysis revealed unexpected similarities in the dark current characteristics of the MAPI and CPB materials. Despite the larger bandgap of CPB (2.3 eV) compared to MAPI (1.5 eV), which theoretically should result in lower dark currents for CPB, both materials exhibited comparable dark current values. MAPI-based detectors showed dark currents in the range of 1–100 nA/cm^2^, depending on the specific detector structure, similar to the results shown in [17]. Surprisingly, CPB-based detectors demonstrated similar values, contrary to our initial expectations. We hypothesize that one reason for this similarity is a slightly higher concentration of electrically active defects (e.g., traps at grain boundaries, grain surfaces within the bulk film, and layer interfaces) in the CPB sensor compared to the MAPI sensor. Trap-assisted conductivity, driven by an optimal trap density, has been linked to photoconductive gain in perovskite-based X-ray detectors and may also account for the higher dark current observed in the CPB-based detectors here. Another reason for this unexpected parity in dark current performance may be attributed to the unique interactions between these perovskite materials and the polymer-based hole-transporting layers used in our detector designs. These interactions could be moderating the flow of charges through the detector, leading to comparable dark current characteristics despite their differing bandgaps. This finding highlights the complex interplay between perovskite active layers and charge transport interfaces in determining overall detector performance.

**FIGURE 1 |:**
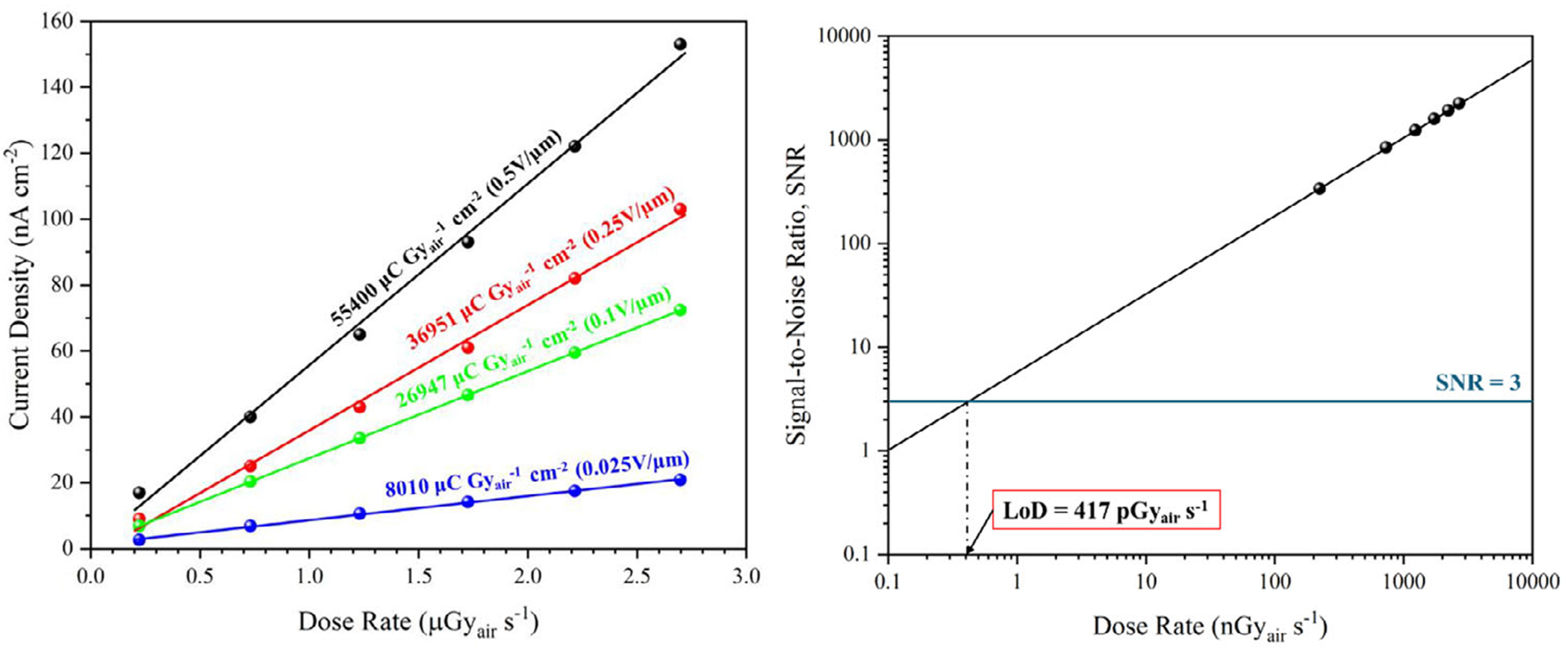
(Left) Current density versus low dose rate at different applied electric fields (0.025 to 0.5 V/μm) for CPB X-ray detectors. The linear relationship demonstrates consistent detector response, with sensitivity (slope) increasing with increasing electric field. (Right) Limit of detection (LoD) characterization for the CPB-based detector at 30 keV and 0.5 V/μm. The LoD was estimated to be 417 pGy_air_ s^−1^ by using the reverse extension line to the point where the Signal-to-Noise Ratio (SNR) = 3. Calculations of the SNR and LoD were performed according to the procedure outlined by Jin et al. [42].

In terms of X-ray sensitivity, the CPB-based detectors demonstrated remarkable performance. We conducted a detailed analysis of the CPB and MAPI detector’s photocurrent response to 30 keV X-rays across a range of low dose rates from 0.22 to 2.70 μGy_air_ s^−1^. The minimum experimental dose was limited by the X-ray source stability and not the sensor performance. The results revealed a highly linear relationship between photocurrent and dose rate for both CPB and MAPI, a critical feature for accurate X-ray detection and imaging. At an applied electric field of 0.5 V/μm, the CPB detector achieved an impressive sensitivity of 55400 μC Gy_air_^−1^ cm^−2^ and an extremely low limit of detection of 417 pGy_air_ s^−1^ (Figure 1). For comparison, our MAPI-based detectors achieved a maximum sensitivity of 13500 μC Gy_air_^−1^ cm^−2^, which represents strong performance for hybrid perovskite systems. It should be noted that the maximum sensitivities we observed here for CPB-based and MAPI-based detectors are higher by about two orders of magnitude when compared to their theoretical maximum sensitivities of 327 μC Gy_air_^−1^ cm^−2^ and 278 μC Gy_air_^−1^ cm^−2^ (calculated using a model similar to those from Girolami et al. [24], Tsai et al. [26], and Pan et al. [41]), respectively. This again suggests the presence of trap-assisted photoconductive gain within our perovskite-based detectors.

The voltage dependence of the detectors’ sensitivity was particularly noteworthy. For CPB, we observed a significant increase in sensitivity with increasing electric field, with values rising from 8010 μC Gy_air_^−1^ cm^−2^ at 0.025 V/μm to 55400 μC Gy_air_^−1^ cm^−2^ at 0.5 V/μm. MAPI detectors showed a similar trend but with lower overall sensitivities, ranging from 7500 to 13500 μC Gy_air_^−1^ cm^−2^ for applied electric fields of 0.25–0.5 V/μm for a 200 μm-thick detector.

This more than sixfold increase in sensitivity for CPB suggests that higher electric fields substantially improve charge collection in the material, an effect attributed to several factors. First, the increased electric field strength reduces the probability of electron–hole recombination by more rapidly separating the photogenerated charge carriers. Second, it may lead to field-enhanced mobility, a phenomenon observed in some semiconductors in which the carrier velocity increases nonlinearly with the electric field strength. Additionally, the higher field could assist in overcoming potential barriers at grain boundaries in the polycrystalline material, facilitating more efficient inter-grain charge transport. The field dependence also suggests the presence of shallow traps in the material; at higher fields, carriers can more readily escape these traps, leading to improved charge-collection efficiency.

The enhanced charge-collection efficiency observed in CPB-based detectors can be attributed to the inherent properties of all-inorganic MHPs. These materials demonstrate better suppression of ion migration than their hybrid counterparts, such as MAPI. In the case of CPB, the inorganic cesium cation and the bromide halide are less mobile than the organic methylammonium cation and iodide halide in MAPI, respectively, leading to reduced ion migration under applied electric fields [29, 30]. This reduced ion migration has several beneficial effects on detector performance. It minimizes the formation of space charge regions within the material, which can distort the internal electric field and impede charge collection. It also reduces the likelihood of defect formation and material degradation over time, contributing to better long-term stability.

We estimated the mobility-lifetime (μ*τ*) characteristics for the CPB sensors using the classical Hecht equation (Equation (1)).

(1) $$I=I_{0}\frac{\mu \tau E}{d}\left[1-exp\left(-\frac{d}{\mu \tau E}\right)\right]$$

where *I_0_* is the saturated current, *d* is the thickness of the MHP film, and *E* is the electric field across the film.

**FIGURE 2 |:**
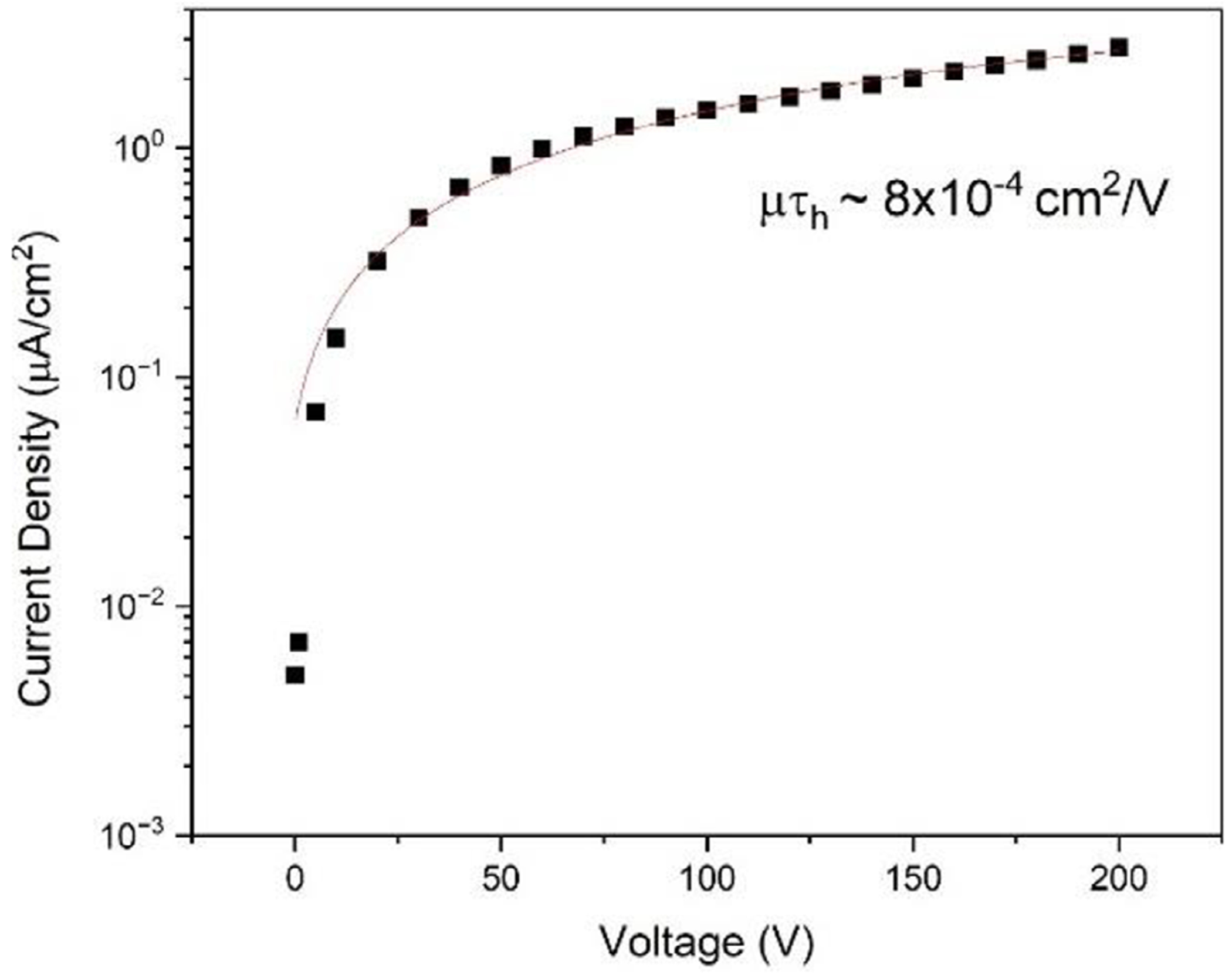
Photoconductivity response of a CPB-based X-ray detector under varying applied biases. The current density (μA/cm^2^) is plotted on a logarithmic scale against the applied voltage (0–200 V) under 30 kVp X-ray irradiation. The non-linear response demonstrates the voltage-dependent charge collection efficiency of the detector. From this data, the μ*τ* product for holes in the CPB sensor was calculated to be 8.0 × 10^−4^ cm^2^/V, indicating efficient charge transport within the material. This relatively high μ*τ* value contributes to the detector’s excellent sensitivity and contrast performance.

For the 200 μm-thick polycrystalline MAPI, the μ*τ* product for holes was found to be 1.3 × 10^−4^ cm^2^/V. A detailed calculation is shown in reference [17]. These values are comparable to those of 250 μm-thick polycrystalline HgI_2_ sensors and are three orders of magnitude better than those of polycrystalline CZT. In our current study, we found the μ*τ* product for holes in CPB to be 8.0 × 10^−4^ cm^2^/V (Figure 2). These relatively high μ*τ* products are indicative of the excellent charge transport properties of the MHPs, suggesting that the charge carriers can travel considerable distances before recombining or being trapped, which is crucial for efficient charge collection in thick detector layers. The higher μ*τ* product observed for CPB aligns with its high field-dependent sensitivity characteristics. The strong field dependence itself implies that the μ*τ* product for CPB is higher than that for MAPI as the charge collection efficiency shows a more dramatic improvement with increasing field. A possible reason for the higher μ*τ* product for CPB could be attributed to the more ordered crystal structure of the all-inorganic perovskite, which may provide more direct conduction pathways for charge carriers. Additionally, the precursor material that was used to prepare the CPB films was derived from ultrahigh purity spectroscopic-grade CPB single crystals (Figures S2–S5) with ppm level impurities whose μ*τ* product for holes was calculated to be 1.7 × 10^−2^ cm^2^/V [40]. The high μ*τ* products of both materials, but particularly CPB, have significant implications for detector design and performance. They enable the fabrication of thicker detection layers, thereby increasing X-ray stopping power without severely compromising charge collection efficiency. This is particularly advantageous for higher energy X-ray detection, where thicker sensors are required to achieve adequate absorption.

Furthermore, as discussed earlier, the combination of high μ*τ* products and high X-ray stopping power in CPB addresses one of the key limitations of current commercial direct conversion materials like a-Se. Perovskite detectors, particularly those based on CPB, show promise for extending the benefits of direct conversion (high spatial resolution, good contrast) to higher energy applications such as chest radiography or computed tomography. The superior performance of CPB at higher fields also indicates its potential for use in applications requiring high sensitivity, such as real-time X-ray imaging or high-flux synchrotron experiments.

**FIGURE 3 |:**
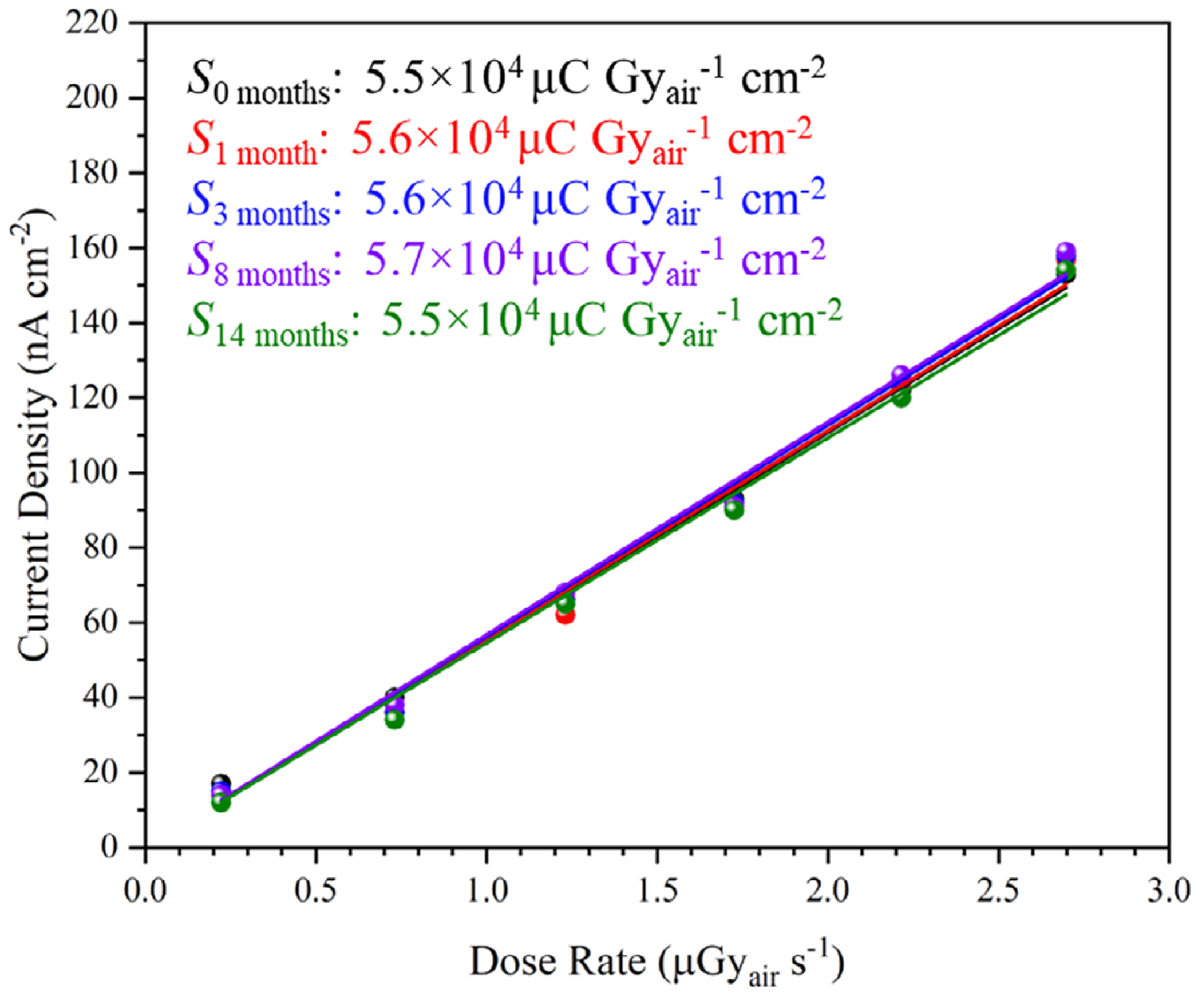
Current Density as a function of Dose Rate plots for a CPB-based detector. The slope of each line gives the sensitivity of the detector. Notably, the sensitivity of the CPB-based detector did not decrease over a 14-month (420-day) testing period. Note that the unencapsulated CPB-based detector was stored in air and in the dark between measurements.

The suppression of ion migration not only contributes to the higher sensitivities observed but also suggests potential advantages in terms of long-term stability and performance consistency. In hybrid perovskites like MAPI, ion migration can lead to the formation of trap states and the segregation of halide ions, potentially degrading performance over time. However, our long-term stability studies on MAPI detectors revealed some surprising and encouraging results. We conducted an extensive 240-day stability study on five MAPI detectors encapsulated in glass and edge sealed epoxy. These detectors were kept under constant bias voltage throughout the study period. Contrary to initial concerns about ion migration-induced degradation, we observed remarkable stability in the dark current baseline of these detectors. In fact, the dark current showed a favorable trend, decreasing from an initial value of 1.29 × 10^−6^ mA/cm^2^ to approximately 2.5 × 10^−7^ mA/cm^2^ over the 240-day period. This reduction in dark current over time is a positive outcome, as it suggests that the detector’s noise characteristics improve with extended operation. Even more impressively, the X-ray sensitivity of the MAPI detectors remained highly stable throughout the study. We observed excellent stability in both systems, with sensitivity variations of less than ±4% over the entire 240-day period for MAPI detectors and less than ±2% over the entire 420-day period for CPB detectors (Figure 3), both demonstrating reliability for long-term applications. This level of consistency is crucial for field applications, as it ensures reliable and reproducible measurements over extended periods without the need for frequent recalibration.

From a practical product development perspective, it is crucial to consider the real-world applications and challenges X-ray detectors may encounter. Commercial or medical settings often involve frequent temperature fluctuations (for example, during shipping), varying humidity levels, and other environmental changes that could potentially stress the detector materials and the encapsulation. Relying heavily on encapsulation to ensure stability introduces an additional layer of complexity and potential points of failure. Any compromise in the encapsulation integrity could lead to rapid degradation of the hybrid perovskite material (MAPI), potentially resulting in unreliable performance or device failure. In contrast, fully inorganic perovskite detectors, such as CPB, offer inherent advantages in stability and resilience. Their all-inorganic nature makes them inherently less susceptible to environmental degradation, reducing the critical dependence on perfect encapsulation. This robustness translates into several practical benefits, including reduced manufacturing complexity and costs associated with high-performance encapsulation, potentially longer shelf-life and operational life in varied environments, greater tolerance to imperfect handling or minor damage during installation or maintenance, and more consistent performance across a wider range of operating conditions.

Ultimately, given the impressive X-ray detection performance of both MAPI- and CPB-based detectors, we adopted a vertically integrated approach to optimize PeroXIs for each MHP to demonstrate their imaging capabilities. X-ray imaging was accomplished using our custom in-house digital radiography system (see Methods for further details).

### 2.2 | Comparison of M-PeroXI and C-PeroXI Performances

**FIGURE 4 |:**
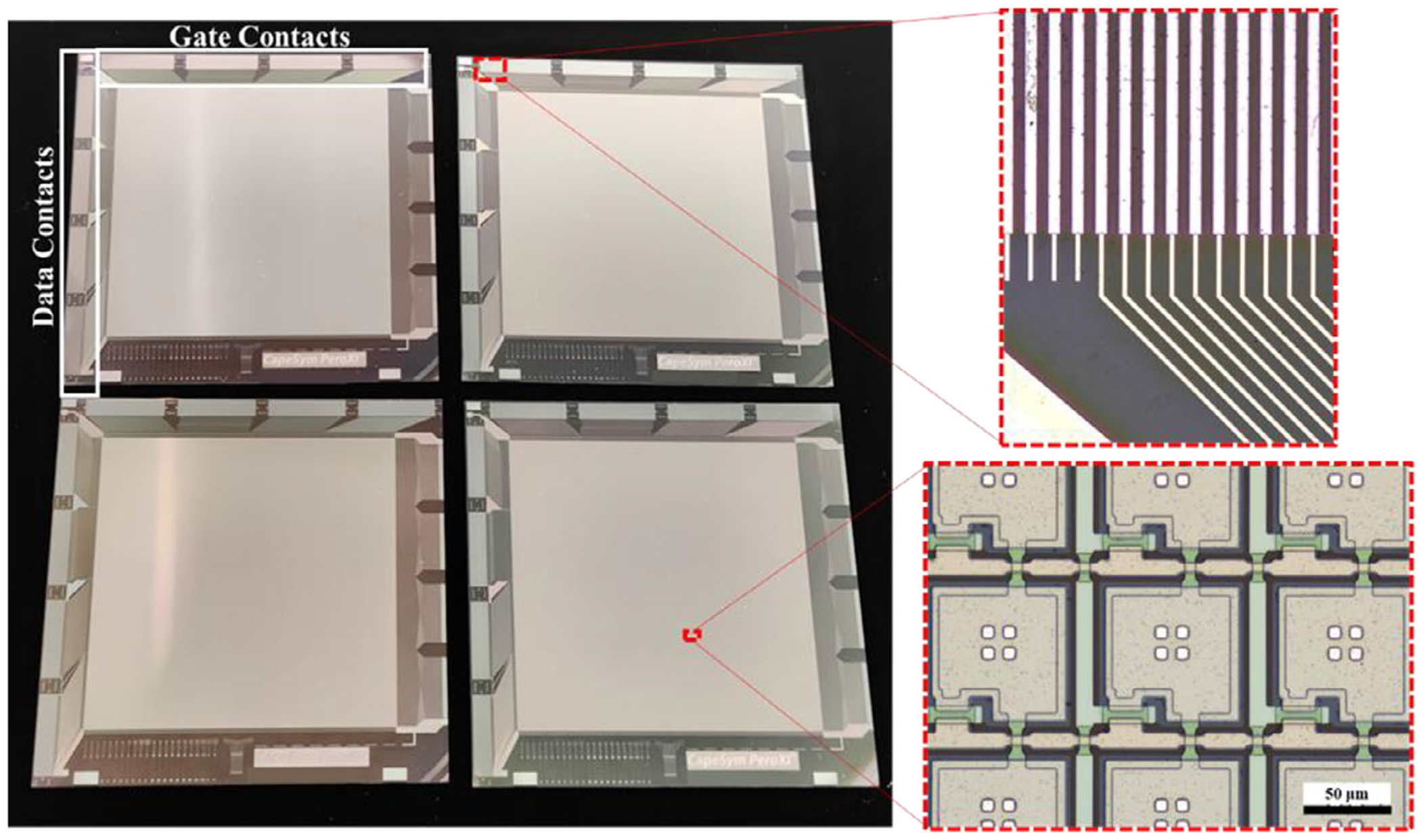
Custom a-Si:H APA backplanes used for fabricating the PeroXIs. The main image shows four identical 2-inch × 2-inch (5.12 cm × 5.12 cm) APA backplanes, each with a 512 × 512 pixel matrix. The Gate and Data contacts are clearly visible along the edges of each backplane. Top-right inset: Magnified view of the Gate and Data contact lines, illustrating the intricate interconnect structure that addresses individual pixels. Bottom-right inset: High-resolution laser microscopy image of the pixel array, showing the detailed structure of individual pixels. The scale bar indicates 50 μm, highlighting the 100 μm pixel pitch of the array. These custom-designed APAs feature amorphous silicon TFTs for each pixel, enabling direct integration with the perovskite sensor layers for high-performance X-ray imaging.

For fabricating the PeroXI detectors, we used a custom APA backplane with a 2 inch × 2 inch (5.12 cm × 5.12 cm) active area (Figure 4). This APA featured 512 × 512 pixels with a pitch of 100 μm, specifically designed for direct X-ray imaging with high-Z materials like CPB and MAPI. The APA utilized amorphous silicon (a-Si:H) TFTs for matrix addressing, with each pixel containing an a-Si:H TFT switch (width/length ratio of 1.5) connected to a 0.4 pF storage capacitor. This TFT design offered low off-state current (< 1 pA) and sufficient on-state current for rapid readout, crucial for minimizing image lag and enabling real-time imaging. The metal contact to the semiconductor layer occupied about 80% of the pixel area, with a 10 μm gap between adjacent pixels, optimizing the fill factor while maintaining good isolation. This high fill factor significantly improved X-ray sensitivity compared to conventional designs. Gate address lines ran horizontally and data lines vertically in the array layout, with careful design to minimize parasitic capacitances. The data line capacitance was kept below 20 pF, reducing readout noise and allowing for low-dose imaging. The APA structure incorporated a passivation layer between the TFT/address lines and the semiconductor contact, enabling a 3D architecture that maximized sensor area while protecting the underlying circuitry. This design enabled the fabrication of semiconductor sensors with thicknesses up to 1400 μm, as shown in our previous study [17], providing excellent X-ray absorption efficiency even at higher energies (60–100 kVp). The separate bias lines enabled flexible biasing schemes, allowing optimization of charge-collection efficiency while managing leakage currents. Typical operating voltages ranged from 2–200 V depending on the semiconductor material and thickness. The 100 μm pixel pitch struck a balance between high spatial resolution and adequate signal-to-noise ratio. This resolution is suitable for many medical imaging applications, including general radiography and mammography. The APA’s wide dynamic range, enabled by the combination of the TFT switch and storage capacitor, allowed for accurate capture of X-ray intensities spanning over 4 orders of magnitude. Furthermore, the APA design was compatible with large-area manufacturing processes, making it scalable for full-size medical imaging detectors. The use of a-Si:H technology ensured good uniformity across the array, critical for artifact-free imaging. Overall, this custom APA design provided a versatile, high-performance platform for evaluating different high-Z semiconductor materials, with a focus on achieving excellent spatial resolution, high sensitivity, and low noise in direct conversion X-ray imaging.

**FIGURE 5 |:**
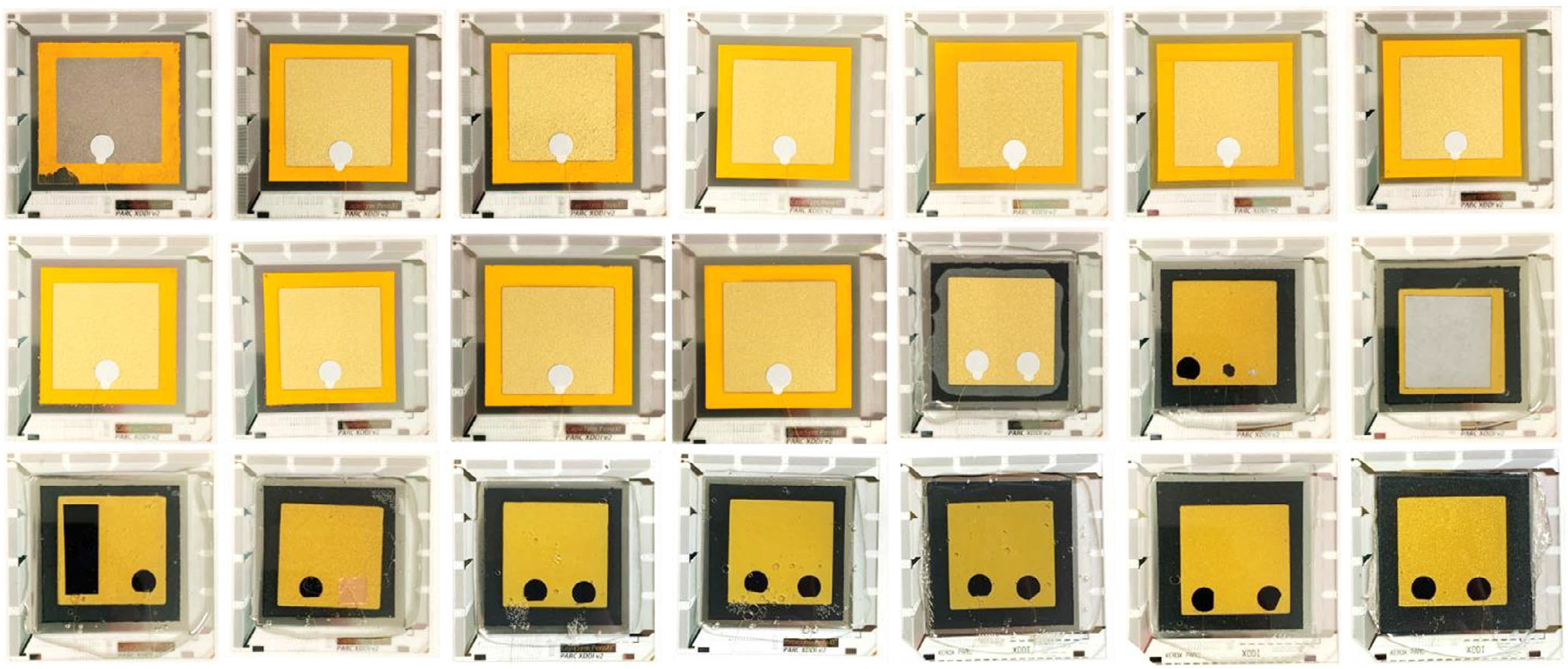
Examples of completed C-PeroXIs (unencapsulated) and M-PeroXIs (encapsulated) were fabricated during this study. The image showcases 21 distinct PeroXIs, illustrating the variety and consistency in fabrication. The top two rows primarily feature C-PeroXI devices, recognizable by their lighter, yellow–orange hue characteristic of CPB. The bottom row displays mostly M-PeroXI devices, identifiable by their darker grey/black coloration typical of MAPI. Variations in color intensity across devices reflect differences in perovskite layer thickness or composition adjustments. The circular and rectangular dark regions on some devices represent different electrode configurations or test structures. This diverse array demonstrates the reproducibility of our fabrication process and the flexibility in device design for both perovskite compositions, underpinning the scalability and robustness of our PeroXI technology.

Despite the advanced features of this APA, we observed some artifacts in our images due to temporary connections between the APA and the readout electronics. This approach was deliberately chosen for several reasons. First, it allowed us to fabricate and test more than 100 detectors (21 of which are shown in Figure 5) without incurring the substantial cost of permanently bonding each detector to the electronics. Second, it enabled us to reuse the same APAs for multiple experiments, significantly reducing material costs and fabrication time. Additionally, this method provided flexibility in testing various semiconductor materials and thicknesses on the same APA, facilitating rapid prototyping and optimization. The temporary connection strategy also allowed for easy troubleshooting and modifications without risking damage to expensive, permanently bonded components. It simplified the process of refurbishing APAs, enabling us to strip off old semiconductor layers and apply new ones for subsequent experiments. While this approach introduced some image artifacts, primarily in the form of occasional line or pixel dropouts due to imperfect contacts, it proved invaluable for our research and development process. These artifacts were well understood and accounted for in our image analysis, ensuring they did not significantly affect our evaluation of the fundamental detector performance.

**FIGURE 6 |:**
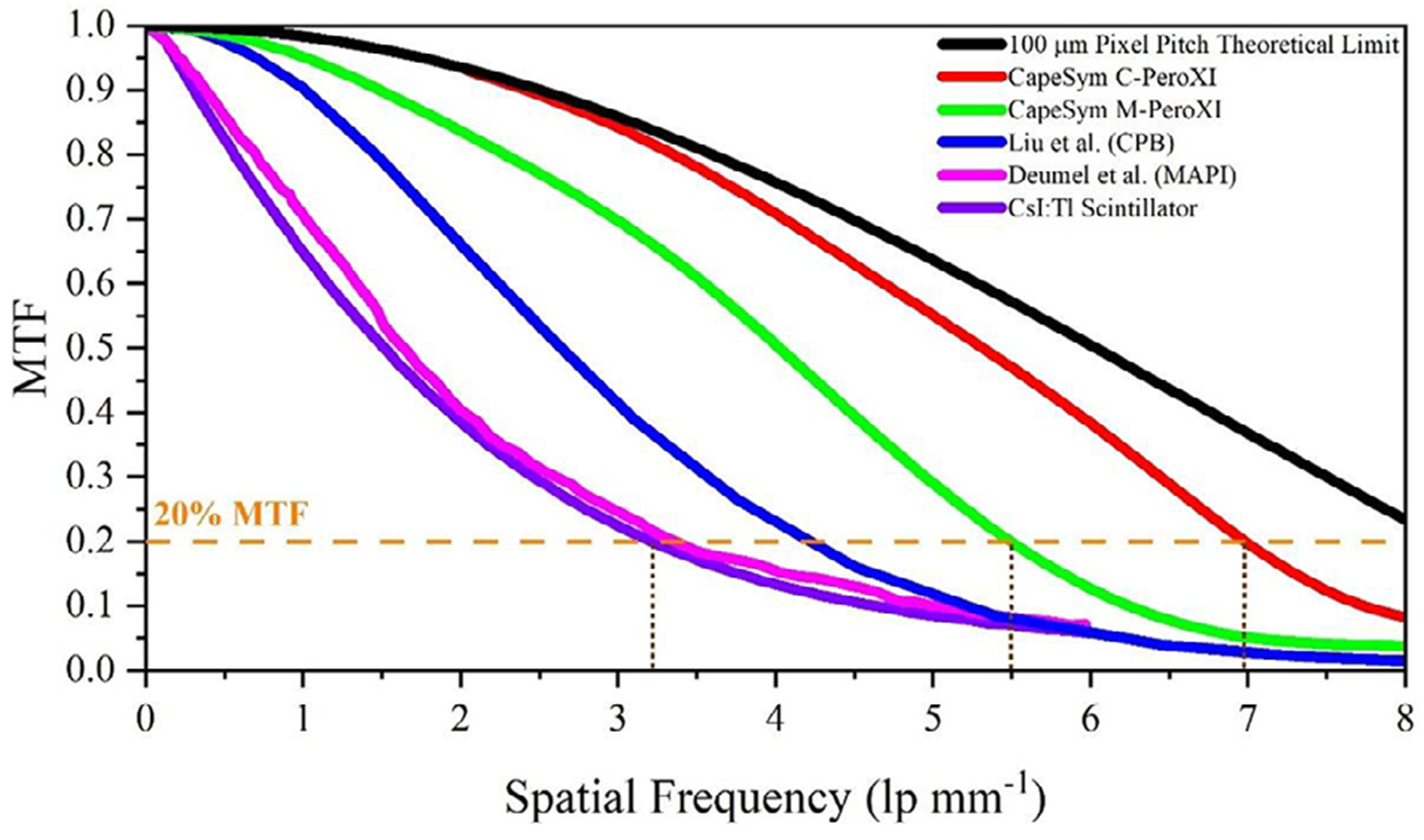
Comparison of MTF responses for various X-ray detectors, illustrating spatial resolution performances. The graph plots MTF against spatial frequency (lp/mm) for different detector technologies. CapeSym C-PeroXI (red) and M-PeroXI (green) from this study, Liu et al. CPB-based FPXI (blue) [34], Deumel et al. MAPI-based FPXI (pink) [39], and conventional CsI:Tl Scintillator-based indirect sensor with same detection efficiency as C-PeroXI (purple). The 100 μm Pixel Pitch Theoretical Limit (black) is the sinc function limit corresponding to the 100 μm pixel pitch of our APA backplane [43]. The dashed orange line indicates the 20% MTF threshold, commonly used to define the practical resolution limit. Vertical dotted lines mark the spatial frequencies at which each detector reaches this 20% MTF threshold. This comparison clearly demonstrates the superior spatial resolution of our C-PeroXI and M-PeroXI devices, particularly the C-PeroXI, which approaches the theoretical limit for the given pixel pitch. It is important to note that the CapeSym C-PeroXI and M-PeroXI utilize a 100 μm pixel pitch, whereas the comparative MAPI-based device by Deumel et al. utilizes a significantly smaller 50 μm pixel pitch [39]. The superior MTF response of the C-PeroXI, despite having pixels twice the size of the Deumel device, indicates minimal crosstalk and superior signal confinement within the optimized CPB sensor layer.

The development of both M-PeroXI and C-PeroXI flat panel X-ray imagers has yielded promising results, each demonstrating unique strengths rooted in their fundamental material properties and optimized device architectures. The M-PeroXI devices achieved a remarkable spatial resolution of 5.5 lp/mm at 20% modulation transfer function (MTF), significantly surpassing the Siemens MAPI-based PeroXI (3.4 lp/mm) [39] and a CsI scintillator (3.2 lp/mm) (Figure 6). This superior resolution can be attributed to the optimization of the MAPI grain structure and the careful engineering of the charge-transport layers. The latter step, in particular, enhanced both contrast and spatial resolution, likely due to improved charge transport and reduced scattering and absorption at interfaces.

C-PeroXIs also demonstrated excellent spatial resolution of 6.9 lp/mm at 20% MTF. As shown in Figure 6, this value approaches the theoretical upper limit of spatial resolution for the APA TFT backplane with a 100 μm pixel pitch used in this study [43]. It is worthwhile to note that the spatial resolution of the C-PeroXI was in line with the theoretical limit up to ≈2 lp/mm, after which it drops off. This is likely due to good alignment between the electric field and the pixel area at spatial frequencies below 2 lp/mm, which then decreases as spatial frequencies increase due to charge sharing. This comparison plot also reveals a counter-intuitive but significant finding regarding detector architecture versus material quality. While the Deumel et al. device features a 50 μm pixel pitch [39]—which theoretically supports a higher Nyquist frequency—our C-PeroXI achieves a significantly higher MTF using a larger 100 μm pixel pitch. Typically, reducing pixel size improves resolution; however, this advantage is lost if the sensor material suffers from high lateral signal spread (crosstalk). The fact that our 100 μm CPB device outperforms a 50 μm MAPI device confirms that our optimized all-inorganic films exhibit superior charge transport properties with negligible inter-pixel crosstalk. This allows the C-PeroXI to maintain high contrast at spatial frequencies where signal spreading degrades the response of the smaller-pixel hybrid device.

Further evaluation of the imaging efficiency of our PeroXI was performed by determining the noise-equivalent dose (NED), detection efficiency (DE), and detection quantum efficiency (DQE(0)) for our C-PeroXI using the procedure outlined in He et al. [38]. Quantifying the NED establishes the intrinsic low-dose limit of the C-PeroXI pixel and enables a direct comparison with state-of-the-art a-Se and CdTe as well as other perovskite imagers. We used the additive noise for calculating the NED by collecting 1000 s of dark current and X-ray dose-generated traces. The time-series was zero-meaned, Hann-windowed, and analyzed with a 4096-point Welch algorithm, yielding a one-sided power-spectral density in A^2^ Hz^−1^. For a frame rate of 1 Hz, we obtained an NED of 52.5 fGy/pixel.

To contextualize the NED, we extracted the detection efficiency (DE) and the quantum-limited ceiling of the detective quantum efficiency from independent material metrics. Monte-Carlo attenuation modelling (predicts a linear attenuation coefficient of 96.5 cm^−1^ for CPB at 30 keV. Charge-transport studies gave a μ*τ* product of 8 × 10^−4^ cm^2^ V^−1^ (Figure 2); at 100 V this translates to a Schubweg μ*τ*E ≈ 4 cm, much higher than the absorber thickness, implying >99% collection efficiency. The product of absorption and collection, therefore, sets an upper-bound DE of ≈ 0.853.

**FIGURE 7 |:**
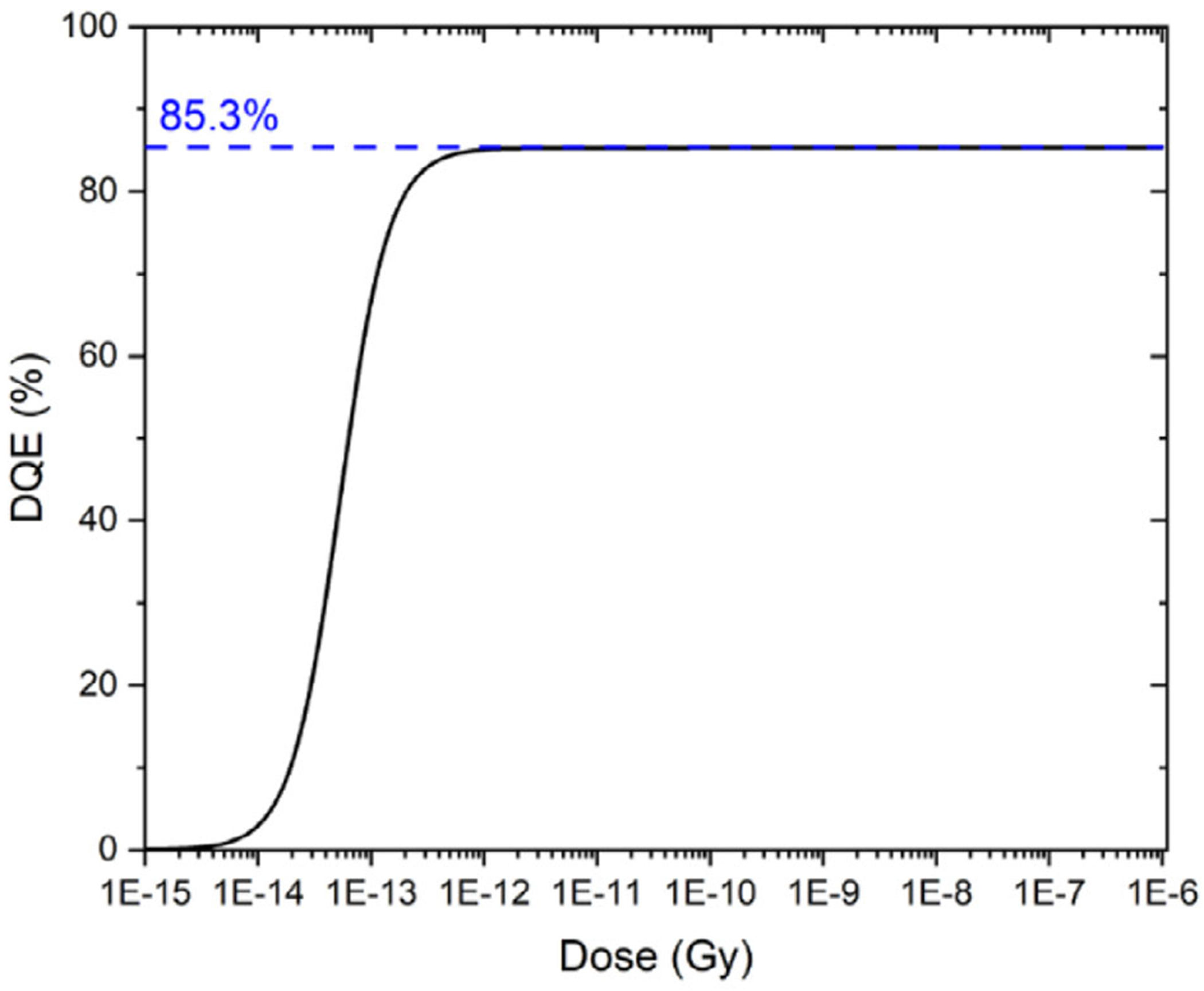
DQE(0)-versus-dose plot, calculated from NED and DE, demonstrating the low-dose imaging efficiency of our C-PeroXI. At ultralow exposures (≈1 fGy), DQE is < 0.1%, reflecting read-noise domination; around 50 fGy (≈ NED), the efficiency rises sharply, surpassing 40% by 200 fGy; and beyond a few hundred fGy, it plateaus at the material-limited ceiling of ≈85.3%.

From the NED and DE data, we constructed a DQE(0)-versus-dose plot (see Methods for more information on plot construction) as shown in Figure 7. The resulting curve shows a steep S-shaped transition: at ultralow exposures (≈1 fGy) DQE is <0.1%, reflecting read-noise domination; around 50 fGy (≈NED) the efficiency rises sharply, surpassing 40% by 200 fGy; and beyond a few hundred fGy it plateaus at the material-limited ceiling of ≈85%. Practically, this means the detector can deliver near-optimal image quality at patient- or sample-safe micro-gray doses but still suffers a pronounced efficiency roll-off when the incident dose drops much below the NED. Engineering routes to lower that value, either by further reducing electronic noise or boosting DE, would expand the detector’s useful dynamic range into the sub-10 fGy regime and enable even lower-dose imaging without sacrificing detective quantum efficiency. Together, the sub-100 fGy NED and ≈85% DQE(0) demonstrate that inexpensive, solution-processed CPB sensors can significantly enhance the low-dose performance of commercial flat panels, underscoring the suitability of halide-perovskite direct detectors for ultra-low-dose fluoroscopy and dental CBCT [44, 45].

On the other hand, the C-PeroXI devices exhibited exceptional contrast in X-ray imaging, achieving a remarkable contrast-to-noise ratio (CNR) of 442 for a high-contrast target, as calculated using Equation (2).

(2) $$CNR=\frac{|\mathrm{Avg Pixel Values for X ray Image ROI}-\mathrm{Avg Pixel Values for X ray Image Background}|}{\mathrm{Std Pixel Values for Image Background}}$$

where “*Avg*” is the average, “*Std*” is the standard deviation, and “*ROI*” is a region of interest in the X-ray-generated image.

**FIGURE 8 |:**
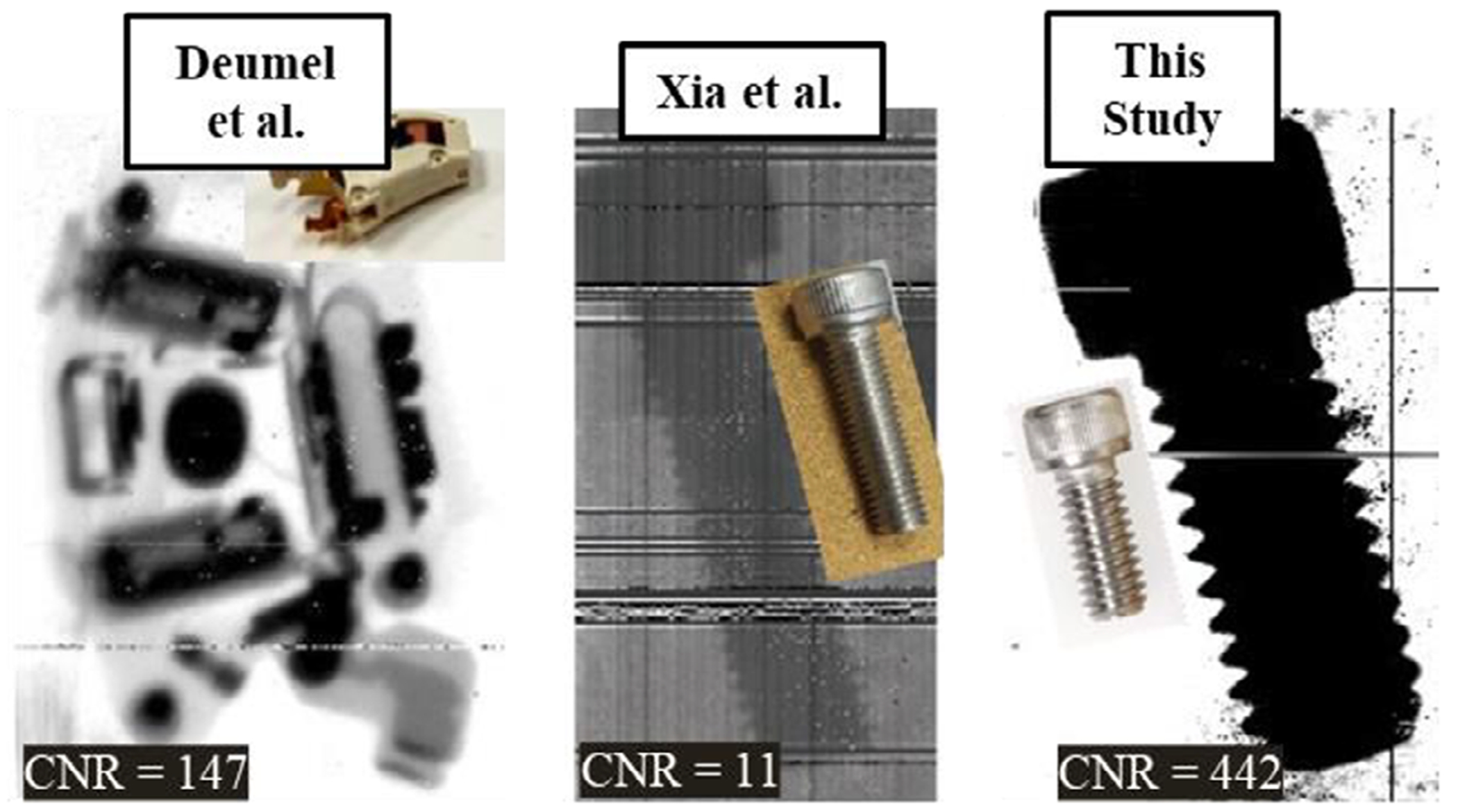
Comparative analysis of CNR in X-ray images from different perovskite-based FPXIs: (Left) X-ray image of a hearing aid captured using a MAPI-based FPXI developed by Deumel et al. [39]. This device features a 50 μm pixel size (half the pixel size used in our study) and achieves a CNR of 147. (Middle) X-ray image of a screw obtained with a MAPI-based FPXI by Xia et al. [30], utilizing a 150 μm pixel size. This detector demonstrates a CNR of 11. (Right) Raw X-ray image of a stainless-steel screw taken with our C-PeroXI (CPB-based) device (50 keV X-ray), featuring a 100 μm pixel size. This imager exhibits a superior CNR of 442. Inset images show photographs of the actual objects being imaged. The CNR values, displayed at the bottom of each X-ray image, quantify the contrast performance of each device. Note that our C-PeroXI achieves the highest CNR despite not having the smallest pixel size, highlighting its exceptional contrast capabilities. All images were acquired under comparable X-ray exposure conditions, emphasizing the superior performance of the C-PeroXI in terms of image quality and contrast sensitivity.

**FIGURE 9 |:**
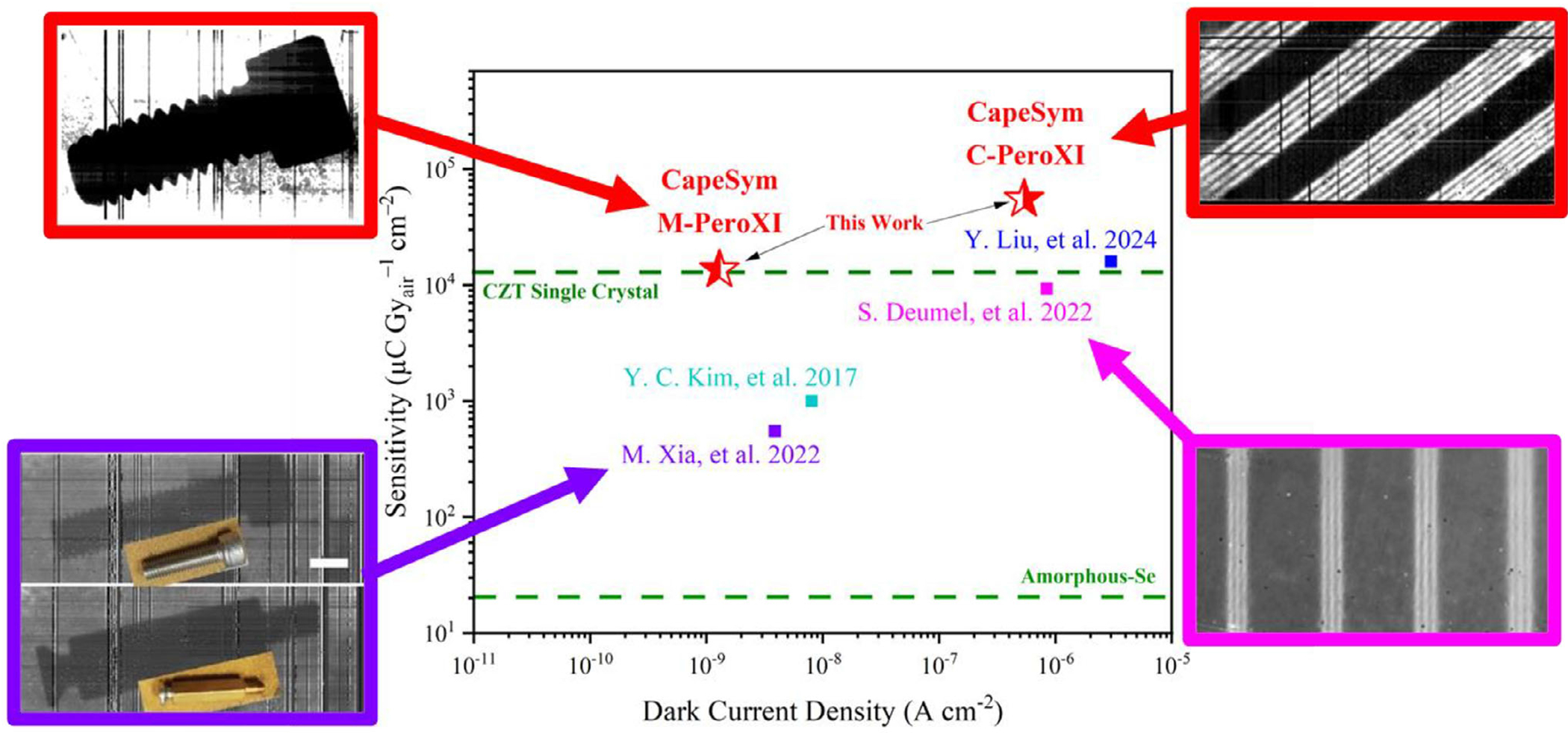
Performance comparison of various X-ray detectors in terms of sensitivity and dark current density. The CapeSym-fabricated C-PeroXI and M-PeroXI (this work, raw images) demonstrate superior performance, combining high sensitivity with low dark current density. These devices outperform previous perovskite-based detectors reported in the literature, including works by Kim et al. [21], Xia et al. [30], Deumel et al. [39], and Liu et al. [34]. The performance approaches and even exceeds that of CZT single crystals while significantly surpassing amorphous-Se detectors. Inset images show representative X-ray images obtained from different detectors, highlighting the improved contrast and resolution achieved by the CapeSym devices.

**FIGURE 10 |:**
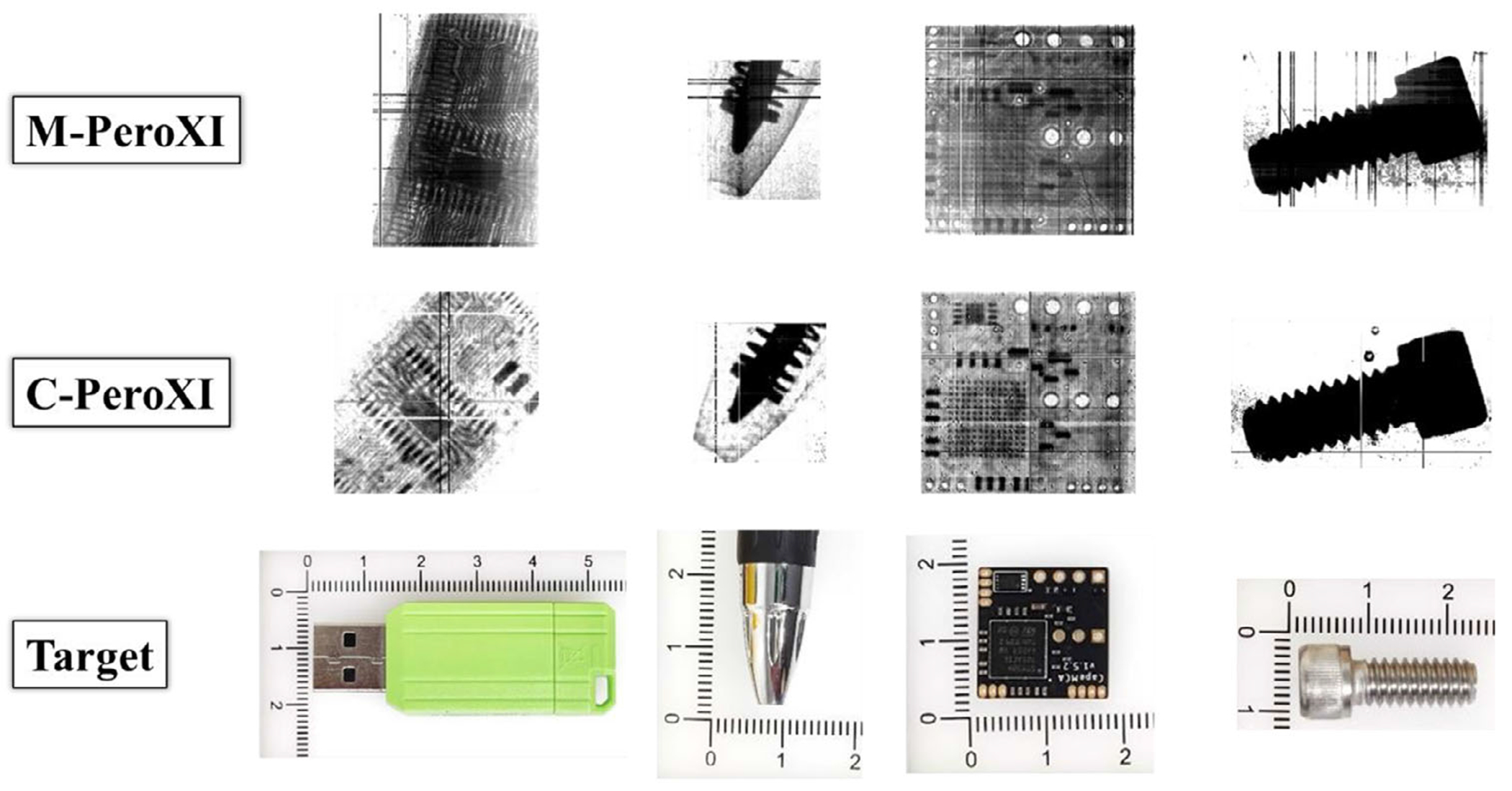
This comparative raw (uncorrected, as acquired using 50 keV X-rays) image set demonstrates the X-ray imaging capabilities of our M-PeroXI (MAPI-based) and C-PeroXI (CPB-based) PeroXIs across a variety of common objects. The sensor thicknesses (Figure S10) and the imaging conditions were kept the same. The top row shows images from the M-PeroXI, the middle row from the C-PeroXI, and the bottom row presents photographs of the actual objects with measurement scales. From left to right, the imaged items are a plastic USB flash drive, a retractable pen, an NXP microchip circuit, and a metal screw. These objects were chosen to showcase the detectors’ performance across different materials and structural complexities. The USB drive and pen images reveal internal components through plastic casings, the microchip demonstrates resolution of fine electronic details, and the screw highlights edge definition and contrast in dense materials. The imaging results show that the C-PeroXI produces images with strong contrast and detail across all tested objects, particularly evident in the intricate structures of the USB drive and the fine circuitry of the microchip.

This significantly outperforms both the Siemens MAPI-based FPXI (CNR of 147) [39] and the Xia et al. MAPI-based FPXI (CNR of 11) [30] (Figure 8). The enhanced performance of the CapeSym C-PeroXI can be attributed to its optimal combination of high sensitivity and low dark current density, as illustrated in Figure 9. The plot shows that the CapeSym C-PeroXI achieves a high sensitivity while maintaining a low dark current density. This combination surpasses other perovskite-based detectors reported in the literature and even exceeds the performance of CZT single crystals, highlighting the exceptional quality of the CPB-based devices. The low dark current contributes to reduced noise, while the high sensitivity ensures strong signal generation, both of which are crucial for achieving high contrast in X-ray images. Also, the excellent contrast of C-PeroXI can be attributed to the inherent stability of the all-inorganic CPB perovskite structure, which likely results in fewer defects and trap states, enhanced charge collection efficiency, and reduced ion migration. To directly compare the imaging capabilities of the M-PeroXI and C-PeroXI, X-ray images of objects with varying contrasts and spatial resolutions were acquired under the same imaging conditions. As shown in Figure 10, both devices successfully revealed internal components of plastic-encased objects like USB flash drives and pens, with the C-PeroXI demonstrating particularly strong contrast and detail resolution of internal electronic components. The C-PeroXI’s superior spatial resolution is further demonstrated by its clearer depiction of fine circuitry in a microchip image. Both devices effectively captured the edge definition and contrast of dense materials in images of a stainless-steel screw. This side-by-side evaluation illustrates the imaging capabilities of both systems, with C-PeroXI showing particularly strong performance in contrast, resolution, and overall clarity that may be advantageous for commercial applications. These improvements can be attributed to the absence of organic components in CPB, unlike in MAPI, which leads to a more robust crystal structure less prone to degradation and ion movement under applied electric fields. In fact, because of these material benefits, the operational stability of the C-PeroXI is exceptional, exhibiting minimal photocurrent drift over 45 min under constant bias and X-ray illumination (Figure S9). This stability contributes to lower image noise, higher contrast, and better spatial resolution, ultimately enhancing image quality and detector stability in the C-PeroXI.

It is noteworthy that these impressive results were achieved despite using APA backplanes with larger pixel sizes (100 μm) compared to some competing technologies (e.g., Siemens’ 50 μm pixels [39]). This suggests that the intrinsic properties of the perovskite materials, combined with optimized device architectures with optimized grain morphologies (Figure S11), can overcome potential limitations imposed by larger pixel sizes. The superior performance of both M-PeroXI and C-PeroXI compared to other perovskite-based FPXIs reported in the literature underscores the potential of solution-processed perovskite materials for next-generation X-ray imaging technologies. The ability to achieve high spatial resolution and excellent contrast using relatively simple fabrication techniques opens up new possibilities for cost-effective, high-performance X-ray detectors in medical, scientific, and industrial applications.

### 2.3 | Comparison with Standard Commercial Detectors

**FIGURE 11 |:**
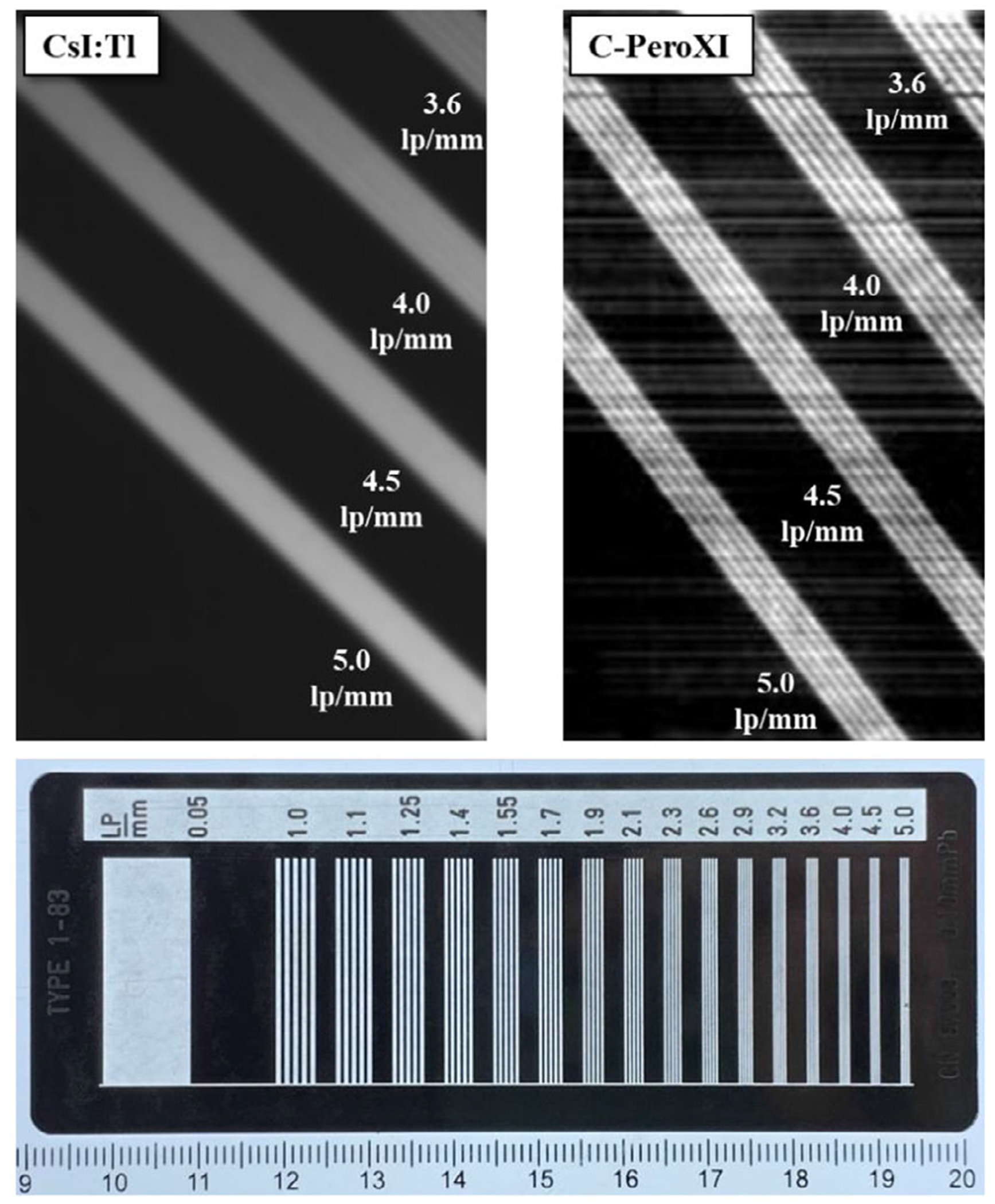
Comparison of raw X-ray images of a line pair pattern target obtained using different detectors of same detection efficiencies. Top left: Image taken with a CsI:Tl scintillator-based indirect detector. Top right: Image taken with direct conversion C-PeroXI with 50 keV X-rays. The C-PeroXI demonstrates superior spatial resolution, clearly resolving line pairs up to 5.0 lp/mm, while the CsI:Tl detector shows blurring at higher spatial frequencies. Bottom: Photograph of the actual line pair pattern target used, showing spatial frequency markings from 0.05 to 5.0 lp/mm. The enhanced contrast and sharpness of the C-FPXI image highlight the advantages of direct conversion perovskite detectors for high-resolution X-ray imaging applications.

The performance of our M-PeroXI and C-PeroXI devices was benchmarked against standard commercial detectors, specifically a CsI-based indirect detector, which is widely used in medical and industrial X-ray imaging applications. Our analysis revealed excellent performance in both spatial resolution and contrast for our perovskite-based direct detectors, underscoring their potential to revolutionize the field of X-ray imaging. At 20% MTF, the M-PeroXI achieved a resolution of 5.5 lp/mm, while the C-PeroXI reached 6.9 lp/mm, significantly outperforming the CsI detector’s 3.2 lp/mm under the same imaging conditions (Figure 11). This marked improvement in spatial resolution can be primarily attributed to the fundamental advantages of direct conversion detectors over indirect detectors.

In direct conversion detectors like our M-PeroXI and C-PeroXI, X-ray photons are directly converted into electrical charges within the perovskite layer. This one-step process eliminates the intermediate step of light generation and subsequent detection present in indirect detectors, which inherently limits their spatial resolution due to light spread. The absence of this light spread in our direct detectors allows for much sharper image formation.

Additionally, the high atomic numbers of elements in our perovskite compositions result in stronger X-ray absorption, leading to more efficient signal generation and potentially lower radiation doses for patients in medical applications.

The contrast performance of the PeroXIs was equally impressive. The C-PeroXI device, in particular, achieved a remarkable contrast-to-noise ratio (CNR) of 442, significantly outperforming the commercial CsI detector, which typically exhibits CNR values in the range of 100–200 under similar imaging conditions. This enhanced contrast is partly due to the direct conversion mechanism, which provides a more linear response to X-ray intensity compared to the logarithmic response of scintillators in indirect detectors. This linearity allows for better differentiation between tissues with similar densities, crucial for accurate medical diagnoses.

Furthermore, our direct conversion detectors do not suffer from the afterglow effects common in scintillator-based indirect detectors. Afterglow can introduce ghosting artifacts and reduce temporal resolution in dynamic imaging scenarios, limitations that our M-PeroXI and C-PeroXI devices overcome. Similar ghosting behavior is also common in a-Se based direct detectors (Figure S12). The absence of a moisture-sensitive scintillator layer also makes our detectors less susceptible to performance degradation over time, potentially extending their operational lifespan. Our perovskite-based detectors offer advantages beyond just resolution and contrast. The ability to achieve these results with solution-processed materials and relatively simple fabrication techniques provides significant benefits in terms of scalability and cost-effectiveness. Moreover, the flexibility in composition and bandgap tuning of perovskite materials opens up possibilities for optimizing detectors for specific energy ranges or imaging applications, a level of customization not easily achievable with traditional scintillator materials.

**FIGURE 12 |:**
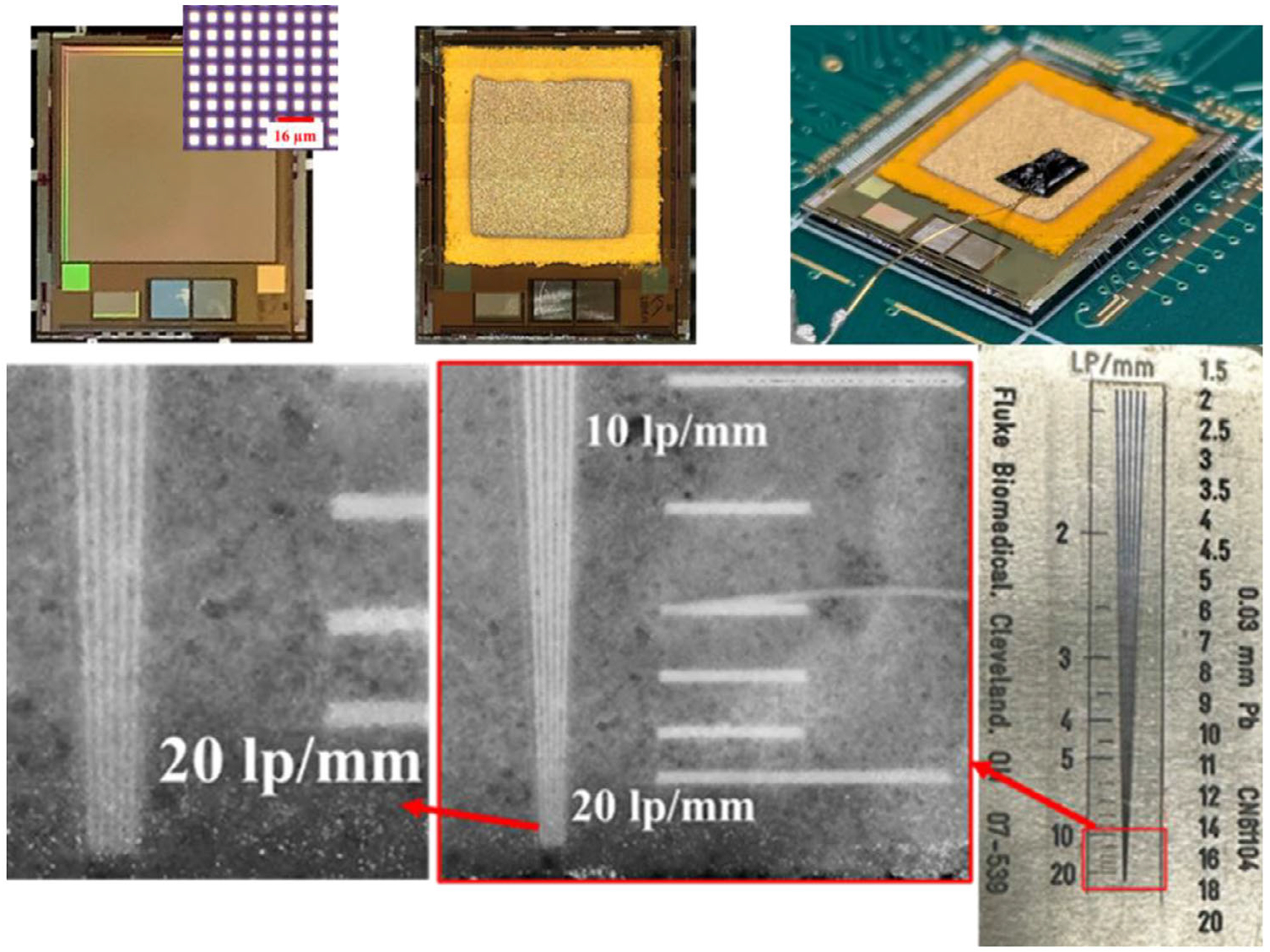
Demonstration of unprecedented spatial resolution achievement in perovskite-based X-ray imaging through high-density pixel integration without implementing any image correction algorithms. (Top) C-PeroXI architecture implementation on a high-density pixel readout: (Left) Image of the third-party 1-megapixel CMOS backplane featuring an industry-leading 7.8 μm pixel pitch, with inset showing the micron-scale pixel array structure. (Middle & Right) Successfully integrated C-PeroXI sensor on the high-density CMOS backplane, demonstrating the future-proof integrability of the perovskite technology to advanced microelectronic platforms. (Bottom) Quantitative validation of the ultrahigh spatial resolution performance without any image correction: (Left) Ultrahigh spatial resolution X-ray image explicitly resolving 20 lp/mm, representing a breakthrough in perovskite detector capabilities. (Center) Expanded field-of-view clearly demonstrating simultaneous resolution of both 10 lp/mm and 20 lp/mm patterns, confirming consistent high-resolution performance across the imaging area. (Right) Standard spatial resolution reference target with red box indicating the analyzed regions, contextualizing this achievement against established imaging metrics.

Additionally, our devices achieved these superior results despite using larger pixel sizes (100 μm) compared to some high-end commercial detectors, suggesting that the intrinsic properties of the perovskite materials, combined with our optimized device architectures, can overcome limitations typically associated with larger pixel sizes. This implies that even higher resolutions could be achieved by reducing pixel sizes, further widening the performance gap between our direct conversion detectors and traditional indirect detectors. To rigorously investigate the fundamental resolution limits of our C-PeroXI technology, we successfully adapted our optimized 200-μm thick CPB detector architecture to interface with a high-performance third-party 1-megapixel CMOS backplane featuring an ultrahigh density 7.8 μm pixel pitch (Figure 12, Top). This represents nearly a 13-fold reduction in pixel dimensions compared to our standard 100 μm pixel pitch design. The detector’s imaging capabilities were evaluated using industry-standard bar pattern phantoms, which revealed remarkable modulation transfer characteristics with distinct resolution of spatial frequencies exceeding 20 lp/mm (or 50 μm) without any image corrections. This achievement is particularly revolutionary as such exceptional spatial resolution has historically been attainable only with thin a-Se sensors, which are fundamentally limited to low-energy X-ray applications due to their poor detection efficiency at higher energies. In stark contrast, our C-PeroXI breakthrough overcomes this long-standing trade-off between spatial resolution and detection efficiency, demonstrating unprecedented high-resolution imaging capabilities even at high X-ray energies. Most significantly, the C-PeroXI maintained robust signal-to-noise performance even at these unprecedented high spatial frequencies, as evidenced by the clearly resolved bar patterns shown in Figure 12 (Bottom). The preservation of strong signal quality at such reduced pixel dimensions provides compelling evidence that our C-PeroXI’s DQE remains fundamentally uncompromised by extreme dimensional scaling. This experimental validation demonstrates that the spatial resolution achieved in our standard 100 μm pixel pitch C-PeroXI configuration is primarily bounded by Nyquist sampling constraints imposed by the pixel size, rather than by intrinsic material limitations such as carrier diffusion lengths or space-charge effects that traditionally restrict resolution in high atomic number semiconductor detectors. This groundbreaking achievement effectively resolves a decades-old challenge in X-ray imaging technology, eliminating the traditional compromise between spatial resolution and detection efficiency that has long constrained the field. The implications are transformative: for the first time, a single detector technology can deliver both ultrahigh spatial resolution and excellent detection efficiency across a broad range of X-ray energies, opening new possibilities for applications from advanced medical imaging to high-precision industrial inspection.

In conclusion, this work elevates solution-processed perovskites from “promising” to category-defining materials for direct X-ray conversion. Our work demonstrates that both MAPI and CPB-based systems offer significant advances in X-ray imaging technology. While both hybrid and all-inorganic perovskite systems demonstrate excellent performance metrics, the inherent environmental stability and reduced ion migration of CPB-based detectors position them as particularly attractive candidates for robust, long-term commercial deployment, despite MAPI having higher X-ray attenuation than CPB. C-PeroXI achieves unprecedented spatial resolution while maintaining exceptional detection efficiency at higher X-ray energies, a combination that has eluded the field for decades and was previously achievable only through mutually exclusive design choices. This revolutionary breakthrough is demonstrated in our all-inorganic CPB-based flat-panel imagers (C-PeroXIs), which not only deliver record detective quantum efficiency (DQE(0) ≈ 85.3%) but, when integrated with a high-density 7.8 μm backplane, achieve unprecedented spatial resolution beyond 20 lp/mm, resolving the long-standing diffraction-and-pixel limits that have constrained this class of detectors. Even on a standard 100 μm-pitch array, the device attains 6.9 lp/mm at 20% MTF and a contrast-to-noise ratio > 440, outclassing commercial CsI:Tl scintillators and rivaling premium CdTe/CZT panels while retaining the scalability and cost advantages of solution processing. Such performance is underpinned by three intrinsic advantages of the fully inorganic lattice: suppressed ion migration, high μ*τ* ≈ 8 × 10^−4^ cm^2^ V^−1^ and robust environmental stability, enabling 14-month, bias-stressed operation with <2% drift, without hermetic encapsulation. Combined with a scalable, low-temperature blade-coating route, these attributes yield a detector that couples 55400 μC Gy_air_^−1^ cm^−2^ sensitivity with a noise-equivalent dose of 52.5 fGy pixel^−1^, establishing the lowest reported dose threshold for any solution-grown perovskite imager. Beyond technical metrics, this work redefines the capabilities of solution-processed semiconductors, paving the way for a new generation of lightweight and affordable imaging systems. Future exploration of material design, device architecture, and large-area integration promises to extend perovskite imagers into domains once thought exclusive to high-cost, crystalline semiconductors, catalyzing a broader transformation across medical, industrial, and scientific imaging.

## 3 | Methods

### Materials

CsPbBr_3_ single crystals were obtained via a procedure outlined in a previous report [40]. MAI (Greatcell Solar, >99.99%), PbI_2_ (TCI America, 99.99%, trace metals basis, [for Perovskite precursor]), GBL (≥ 99%, Sigma–Aldrich), DMF (anhydrous, 99.8%, Sigma–Aldrich), DMSO (anhydrous, ≥ 99.9%, Sigma–Aldrich), NMP (anhydrous, 99.5%, Sigma–Aldrich).

### MAPI and CPB Sensor Fabrication

Either ITO-coated glass (1850 Å thick ITO with a resistivity of 10–15 Ω/sq) substrates (for single-pixel detectors) or a-Si:H active pixel array (APA) TFT backplane (512 × 512; 100 μm pixel pitch) substrates (for PeroXIs) were cleaned via ultrasonication in appropriate solvents and dried with an argon gun. The optimized polymer hole-transporting layer was then deposited on the substrates. A similar strategy was used by Kim et al. [21].

### MAPI:

The MAPI sensor layer was formed from a stoichiometric MAI:PbI_2_ (5N purity) in GBL solution. The solution was prepared following a procedure outlined in a previous report [17]. Deposition of the MAPI sensor material was performed via room temperature blade coating in an inert atmosphere using a film applicator. The coating speed and blade gap were adjusted accordingly to target a MAPI sensor film thickness of 200 μm. After coating, the wet MAPI sensor film was annealed on a hot plate at 90 °C for 1.5 h. Due to the inferior environmental stability of MAPI compared to CPB, all MAPI-based detectors were encapsulated with a thin (0.5 mm) glass slide and edge sealed with epoxy to prevent moisture ingress and degradation while testing. This allowed for a more accurate performance comparison of MAPI-based detectors with CPB-based detectors during X-ray testing in an ambient environment. The encapsulation effects on the X-ray attenuation of the MAPI-based detectors were negligible.

### CPB:

Spectroscopic-grade CPB single crystal, grown using the procedure described in our previous publication [40], was sequentially ball milled until fine CPB nano-powder with particle sizes below 10 nm was achieved. It was found to be extremely important to use a structurally phase pure starting material (orthorhombic single crystalline) for repeatability in results (Figures S2–S5). A CPB nano-powder slurry in DMF and DMSO was used as the blade coating solution. The blade-coated CPB-solvent complex was annealed on a hot plate at 150°C for 2–4 h to achieve a phase-pure orthorhombic CPB sensor film (Figure S6).

For both MAPI- and CPB-based detectors, metal contacts (such as Au, Pb, and Bi) were deposited through appropriately sized shadow masks. Note that all processing conditions employed were done at temperatures equal to or below 200°C to preserve the structural integrity of the APA TFT backplane used to fabricate the PeroXIs. Step-height thickness measurements revealed the thicknesses of the MHP sensor layers to be 200 ± 15 μm (Figure S10). Laser microscopy images of both the sensor film surfaces illustrate the dense and compact nature of the MHP polycrystalline films with grain sizes up to 30 μm (Figure S11).

### X-Ray Imaging and Detector Characterization

Surface profile and morphology measurements were performed with a Keyence VK-X3000 series laser microscope. Scanning electron microscopy (SEM) images for surface and cross-sectional morphology were taken using a JEOL JSM-6300 SEM, operating in an inert atmosphere at an acceleration voltage of 10–15 kV and a current of 10 μA. X-ray diffraction (XRD) data of the perovskite crystals, powders, and films were acquired by a *θ*:2*θ* scan on a Rigaku SmartLab diffractometer equipped with a copper X-ray tube with a Ni k*β* filter, parafocusing (Bragg-Brentano) optics, computer-controlled slits, and a 1D strip detector. X-ray imaging and characterization were performed using digitally controlled microfocus X-ray sources. The X-ray setup consisted of the source, a sample stage, and the detector array, all enclosed in a lead-shielded cabinet. For different dose measurements, the detector-to-source distance was varied appropriately. The dose rates of the X-rays were measured with a calibrated RaySafe Dosimeter. The details of the APA used for imaging are provided in the manuscript. For detector operation, gate voltages were set to 15–20 V for the on-state and −10 to −2 V for the off-state. The readout protocol involved sequentially turning on gate lines one by one, with a typical gate-on time of 10 μs per line. A complete readout cycle consisted of scanning all gate lines to clear pixel charge (reset), X-ray exposure, and then scanning all gate lines again to readout the accumulated charge. The readout timing was controlled by clocking the readout chip. This process was repeated for each gate line, allowing for minimal image lag and efficient charge collection. X-ray sensitivity measurements were performed by varying the X-ray tube voltage (30–90 kVp) and current (10–100 μA) to achieve different exposure rates. The detector response was measured as a function of exposure rate and applied bias voltage to the MHP sensor. Dark current measurements were taken with the X-ray source off to assess detector noise performance. Image quality metrics, including modulation transfer function (MTF) and contrast-to-noise ratio (CNR), were calculated from flat-field and edge phantom images following standard radiographic image analysis techniques. We utilized the standard slanted-edge method (IEC 62220-1 standards) to calculate the pre-sampled MTF. This involves capturing the image of a verified tungsten edge phantom to derive the Edge Spread Function (ESF) and Line Spread Function (LSF). The DQE(0)-versus-dose plot was built by first fixing the one-second-integration constants we had already extracted from the raw data analysis, namely a noise-equivalent dose of NED = 5.25 × 10^−14^ Gy pixel^−1^ and a high-dose detective efficiency of DE = 0.853 (product of quantum absorption and charge-collection efficiency). We evaluated DQE for doses from 1 fGy up to 1 μGy per pixel. All digital analyses were performed using custom scripts.

## Supplementary Material

Supplementary Material

Additional supporting information can be found online in the Supporting Information section.

## Data Availability

The data that support the findings of this study are available from the corresponding author upon reasonable request.
