# Supplementary material for: Performance of Hybrid and All-Inorganic Perovskite Direct X-Ray Imagers: Surpassing Commercial Standards
Source: Adv Mater Technol. Author manuscript; Available in PMC 2026 Jun 24. (PMC13290278; doi:10.1002/admt.202501491)
Supplement: Supplementary Material [file NIHMS2181196-supplement-Supplementary_Material.pdf]

Performance of Hybrid and All-Inorganic Perovskite Direct X-Ray Imagers: Surpassing Commercial Standards

Brandon Dunham[1], Shariar Motakef[1], Amlan Datta[1*]

[1]*CapeSym, Inc., 6 Huron Drive, Natick, MA 01760*

Comparison of MHP and Commercial X-Ray Detector Mass Attenuation Coefficients

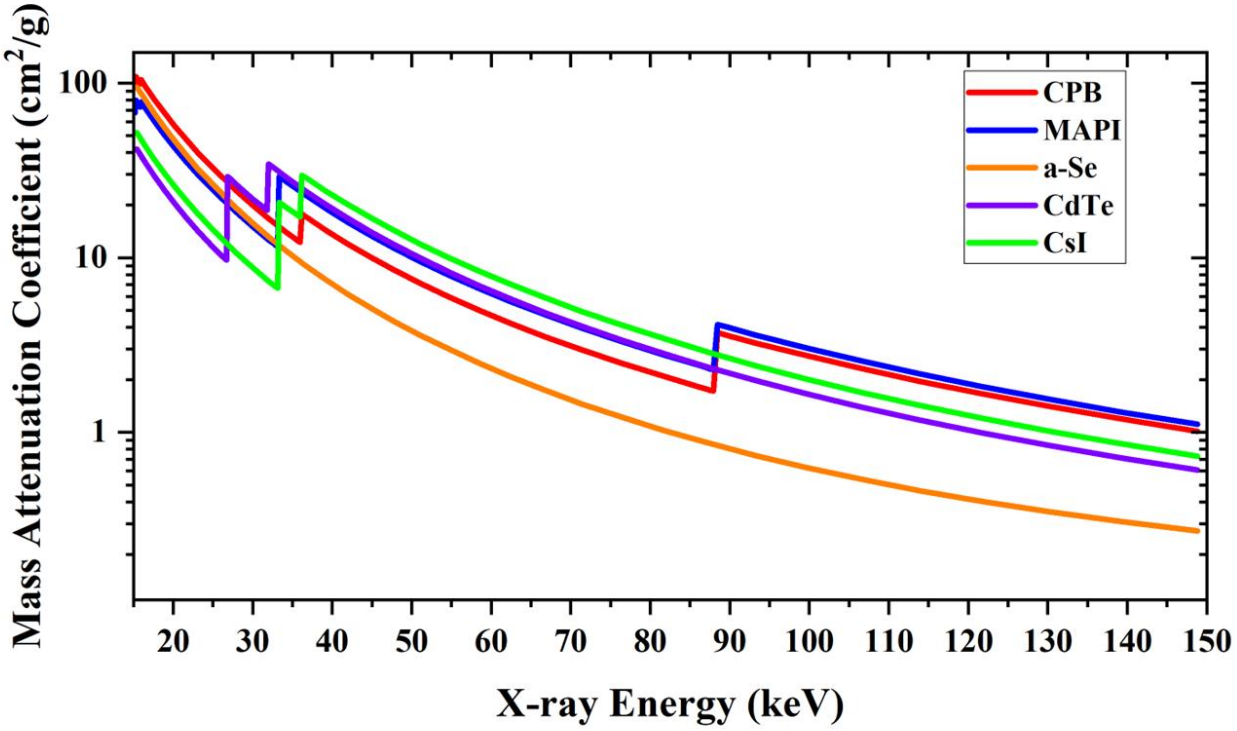

Figure S1. A comparison of the mass attenuation coefficients across different X-ray energies for MAPI and CPB to those for the common commercial X-ray detectors a-Se, CdTe, and CsI. Noticeably, the mass attenuation coefficients for MAPI and CPB are similar to those of these commercial detectors and are occasionally superior to them, specifically at higher X-ray energies >90 keV.

Spectroscopic-grade CPB Crystalline Starting Material

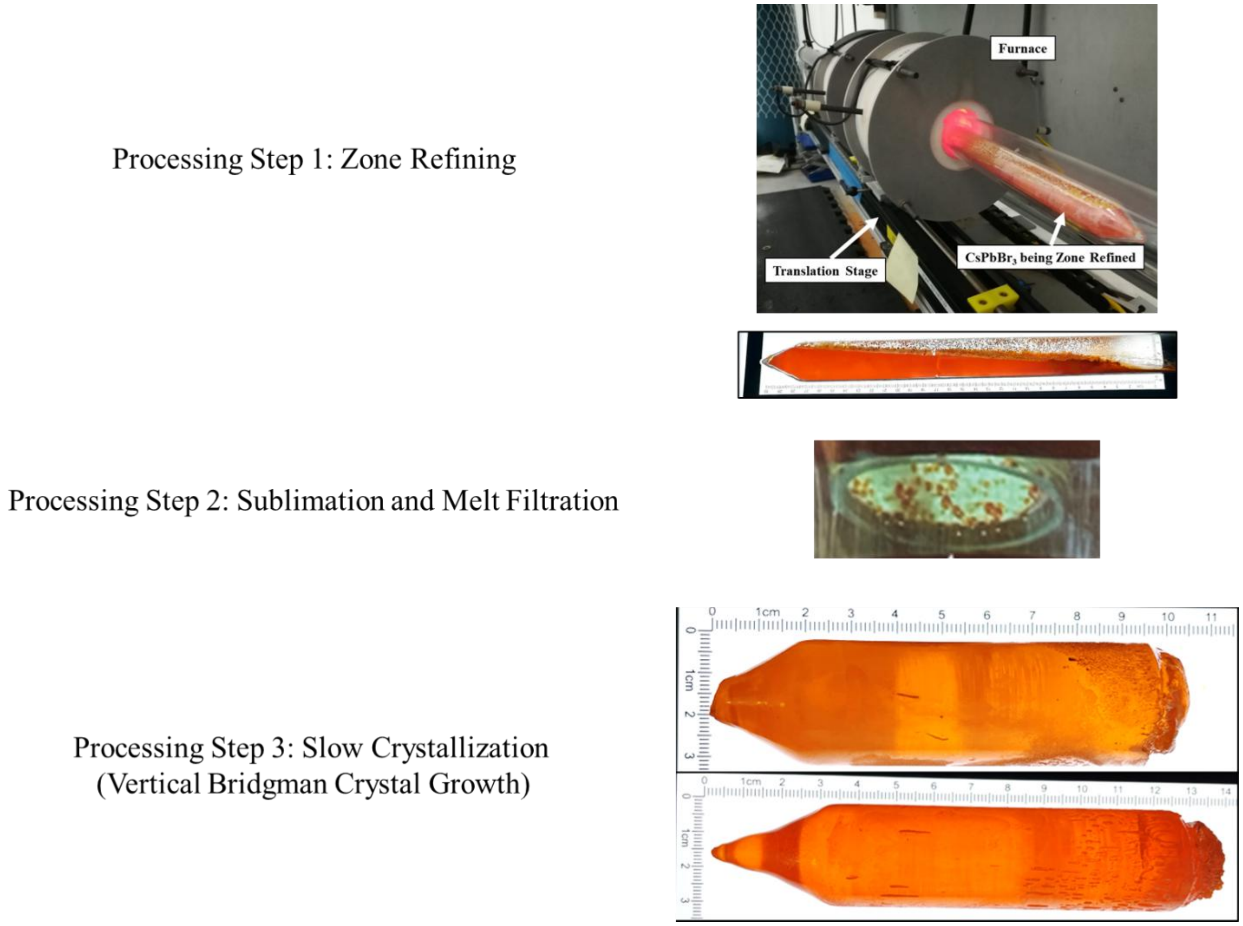

Figure S2. This figure illustrates the three main processing steps in the production of high-quality CPB material that was used as the starting material for the C-PeroXI in this study. Step 1 shows the zone refining process, where a furnace moves along a translation stage to purify the CPB material by segregating impurities. The resulting zone-refined ingot, with its characteristic color gradient, is displayed below. Step 2 depicts the sublimation and melt filtration process, where the purified material is sublimed and then melted and passed through a filter to remove any remaining particulates. Finally, Step 3 presents the slow crystallization phase using the Vertical Bridgman crystal growth method. The main reason for this is to achieve a singular starting phase of CPB for the fabrication of C-PeroXIs to improve repeatability. Two large CPB single crystals, each over 10 cm in length, are shown as the end product of this carefully controlled growth process. These steps collectively ensure the production of high-purity, large-volume CPB material for use in C-PeroXI fabrication.

Glow Discharge Mass Spectrometry (GDMS) Data for Total Impurities in Processed CPB

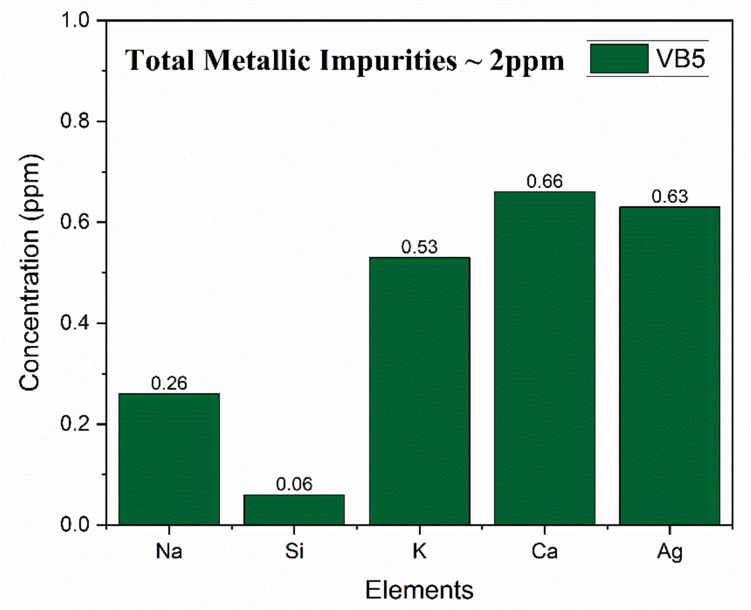

Figure S3. Concentration of metallic impurities detected in the processed CPB material used to fabricate C-PeroXIs. Total metallic impurities were approximately 2 ppm. Si and Ag likely originated from quartz ampoules used in processing, while Na, K and Ca are probable contaminants from handling CPB precursors. The low overall impurity levels demonstrate the effectiveness of the purification methods employed.

Phase Pure CPB Crystal

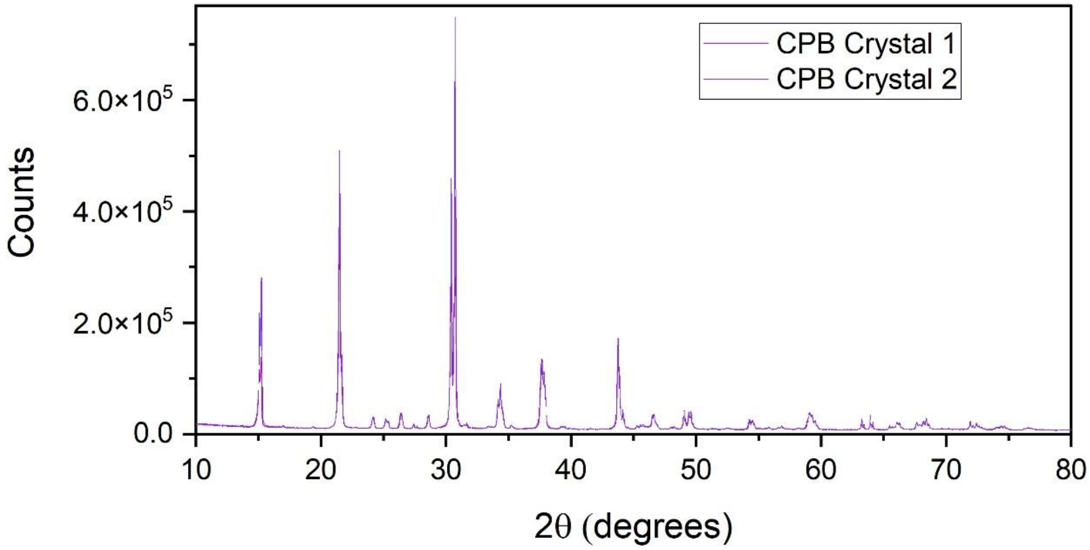

Figure S4. It was found to be extremely important to use phase pure (orthorhombic pnma) CPB as a starting material for the C-PeroXI fabrication to ensure repeatability and good performance. This XRD spectra of the CPB starting material shows representative peaks at approximately 15, 21, 30, 34, 37, and 43 degrees, consistent with literature reports of the stable low-temperature orthorhombic Phase of CsPbBr3 with a pnma space group. Reitveld structural analysis was also performed to determine specific lattice parameters for each sample. All samples have comparable lattice parameters differing by less than 0.1 Å. It was noticed that the $CsPb_2Br_5$ tetragonal phase was present in every starting material that was not grown using the optimized Bridgman technique.

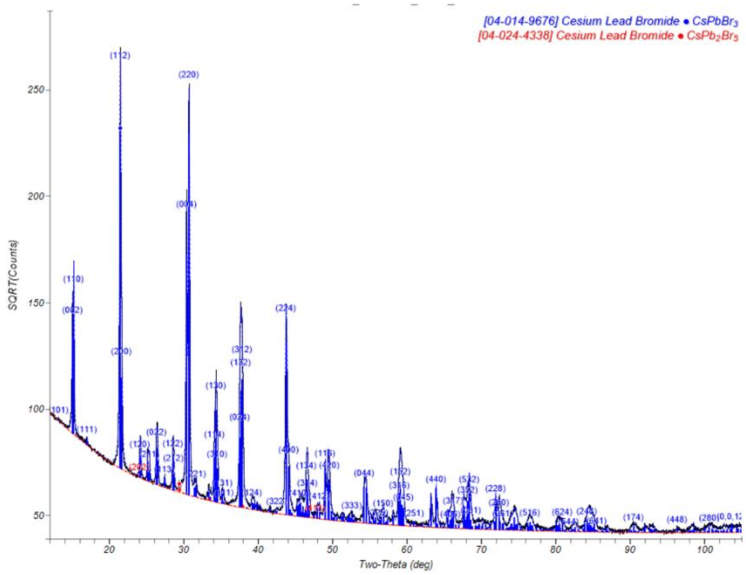

Figure S5. Example of XRD analysis showing the presence of both the orthorhombic and the tetragonal phases in unoptimized CPB starting material.

Phase Pure CPB Sensor Film

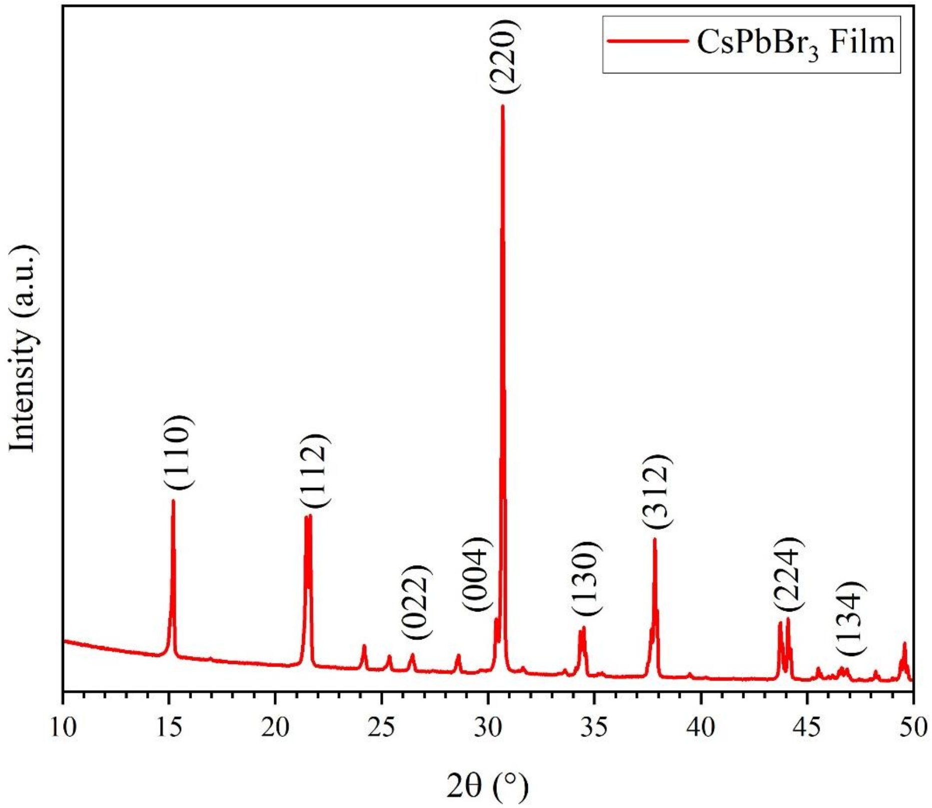

Figure S6. This XRD spectra of the CPB sensor film shows representative peaks at approximately 15, 21, 30, 34, 37, and 43 degrees 2θ, consistent with literature reports of the orthorhombic Phase of $CsPbBr_3$. Peaks corresponding to the $CsPb_2Br_5$ tetragonal phase were not present in these films. The representative peaks for the CPB sensor film matched those present in the phase pure CPB starting material, indicating a desirable crystal-to-powder-to-film transition.

CPB Sensor Precursor Solution and Films Made With Non-Phase Pure Raw Material

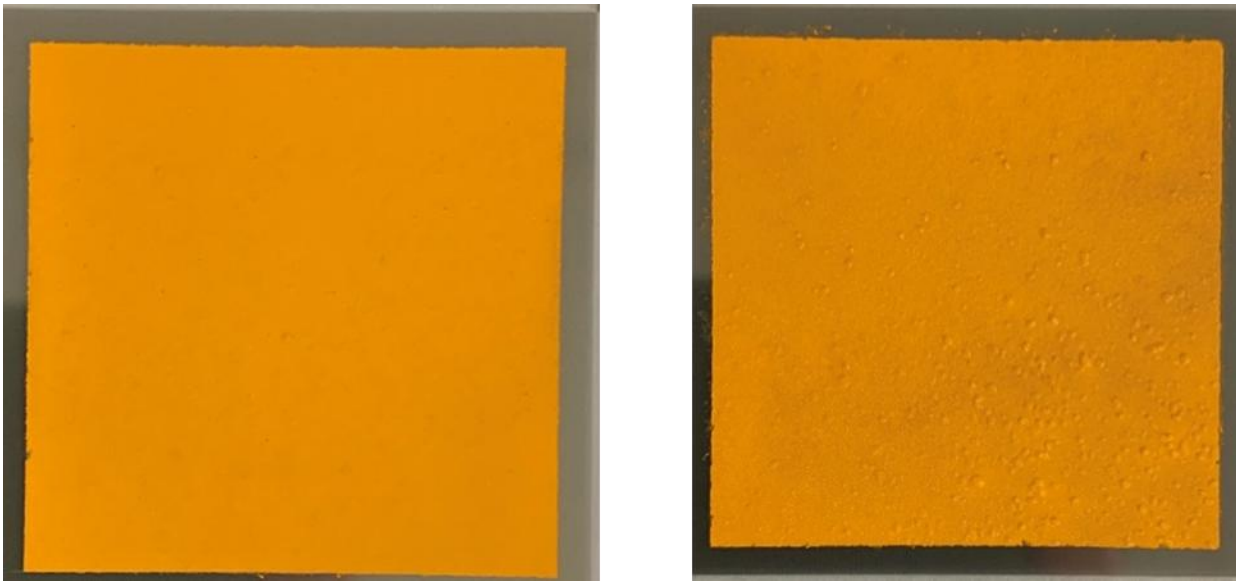

Figure S7. A compact and smooth blade coated CPB sensor film (left) formed from a homogeneous CPB nano-powder solution produced from ultrahigh purity, spectroscopic-grade CPB single crystal precursor materials. A blade coated CPB sensor film with multiple aggregate islands (right) formed from a nonhomogenous CPB nano-powder slurry produced from a CPB single crystal precursor material with traces of the $CsPb_2Br_5$ tetragonal phase and higher levels of metallic impurities.

X-ray Imaging with CPB Sensor Made With Non-Phase Pure Raw Material

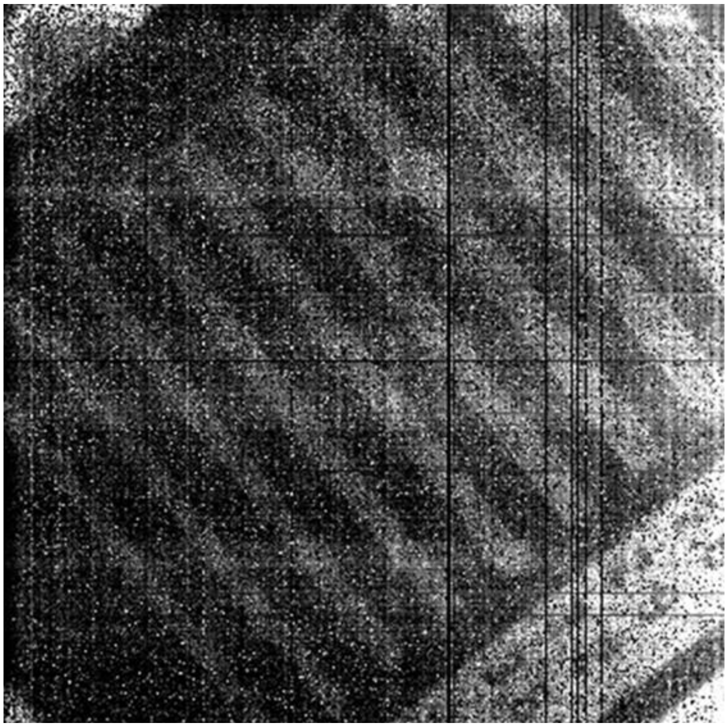

Figure S8. X-ray image of a bar pattern phantom taken using a C-PeroXI with a CPB sensor made from non-phase pure raw material. The CPB sensor filmed was non-homoegenous, had traces of a secondary $CsPb_2Br_5$ tetragonal phase, and contained multiple aggregates. The aggregates and multi-phase film contributed to pixel charge-sharing, which resulted in the blurred X-ray image shown here.

CPB Detector Operational Stability

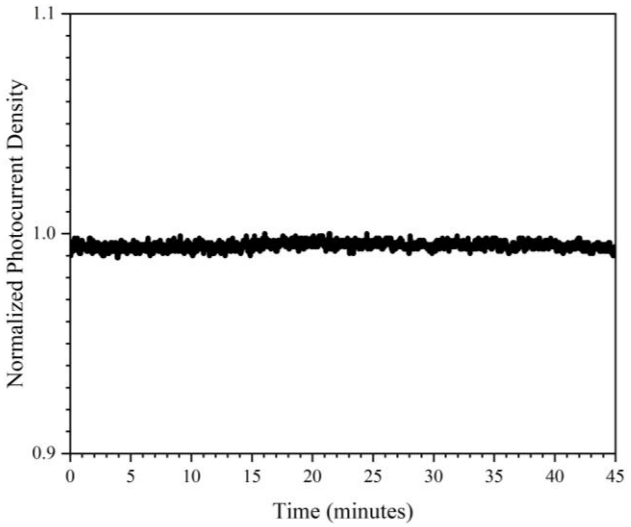

Figure S9. Normalized Photocurrent Density under continuous operational bias and uninterrupted X-ray irradiation for 45 minutes for a C-PeroXI. The minimal photocurrent drift with time implies excellent operational stability under X-ray irradiation.

MAPI and CPB Film Thickness

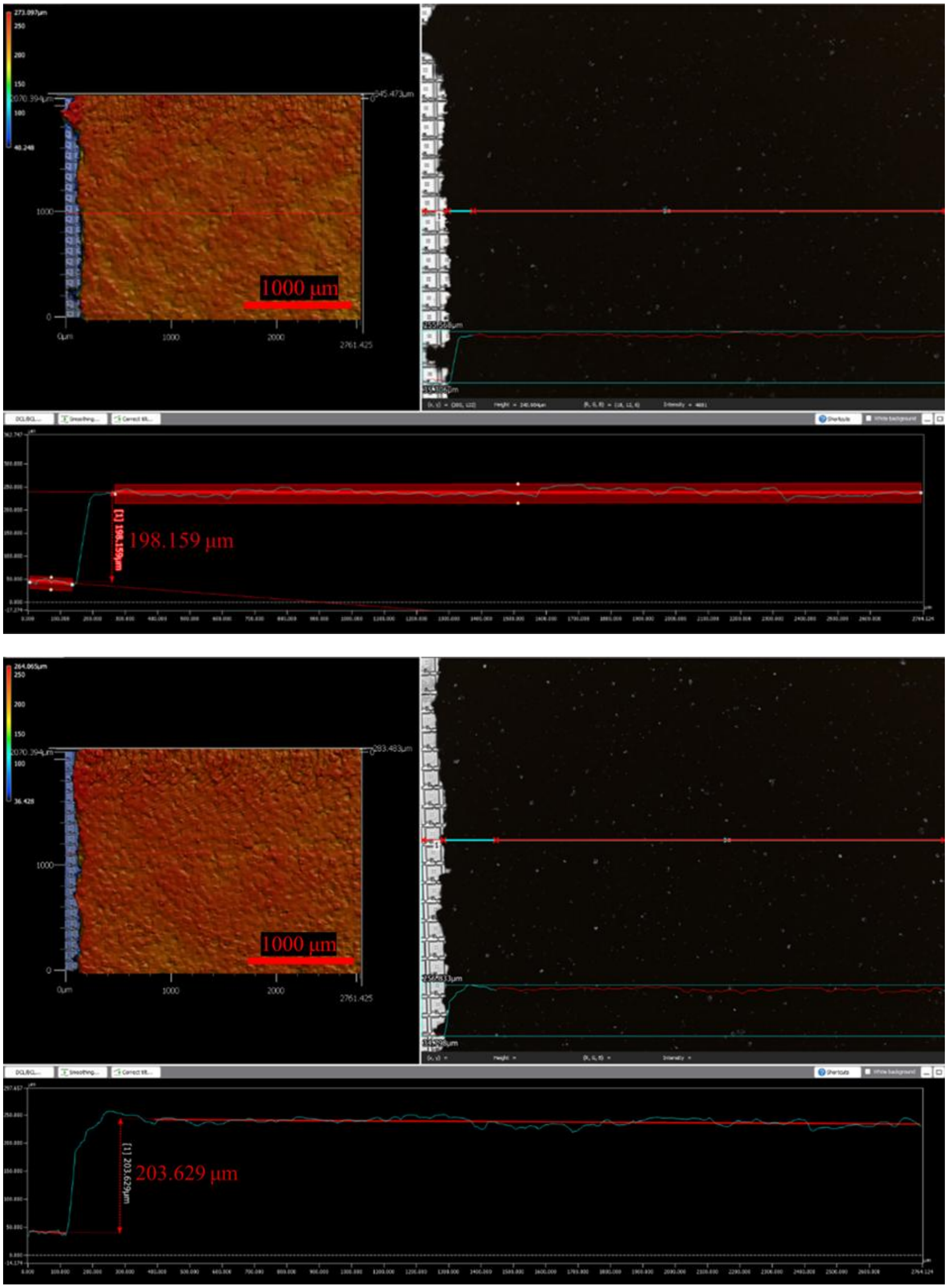

Figure S10. Step-height thickness measurements of a MAPI film (top) and CPB film (bottom) taken with a Keyence VK-X3000 series laser microscope. Step-height thickness measurements reveal the thicknesses of each MHP film to be 200 ± 15 µm.

MAPI and CPB Film Surface Morphology

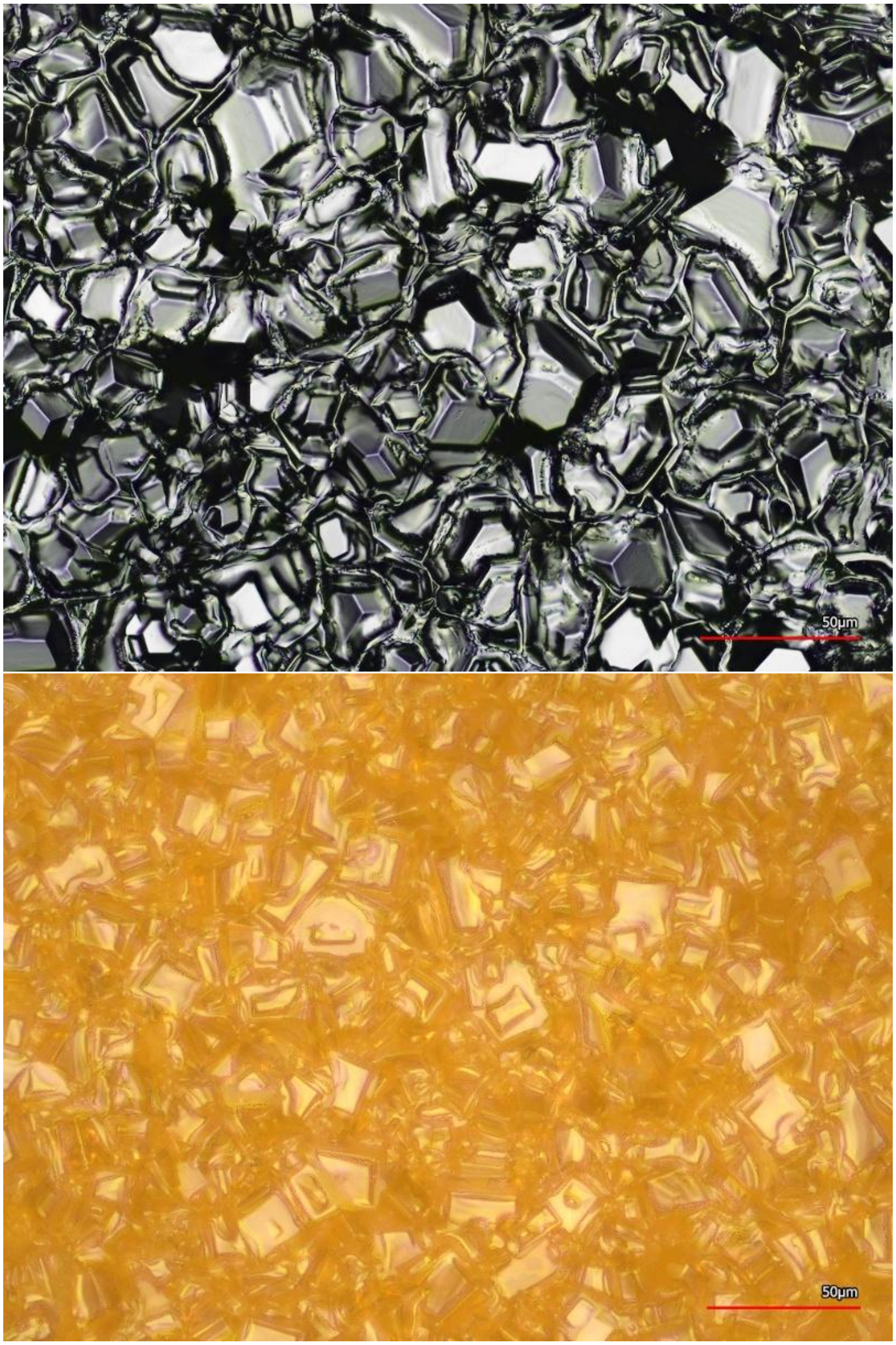

Figure S11. 50x magnification optical images of a MAPI film surface (top) and a CPB film surface (bottom) taken with a Keyence VK-X3000 series laser microscope. Imaging revealed the MHP crystallite grain sizes to be up to ~30 µm and arranged in a dense and compact nature.

Ghosting effect in a-Se Direct Detectors

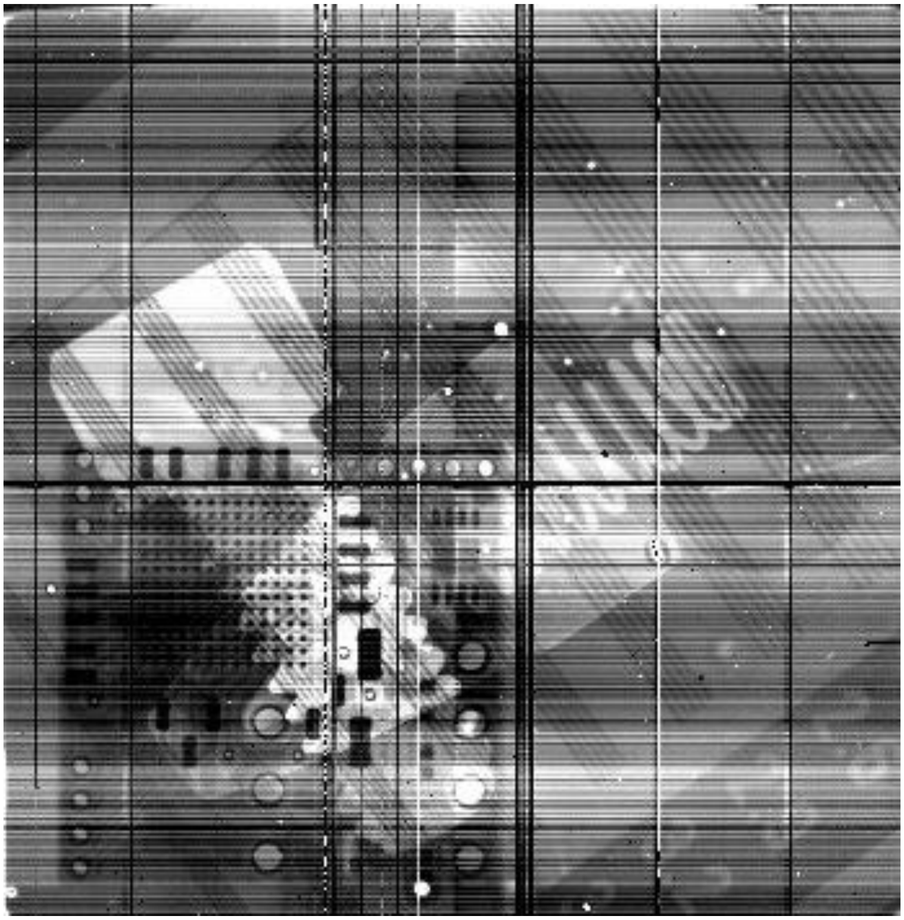

Figure S12. Representative X-ray radiograph acquired with a reference 20-µm-thick a-Se direct FPXI showing pronounced ghosting artifacts. The a-Se sensor layer was deposited on the same a-Si APA backplanes used to evaluate the M-PeroXIs and C-PeroXIs, enabling a direct, backplane-matched comparison. The starting a-Se material was co-doped with As and Cl, according to the optimized recipes reported in the literature. The image contains the same test targets as those used for Figure 10 in the main manuscript; each target was physically replaced within ~30 s between exposures. The observed ghosting is evident as residual contrast and afterimages, as well as non-uniform streaking superimposed on the target features, consistent with lag/charge-trapping–related memory effects.
